# The fate of 35S rRNA genes in the allotetraploid grass *Brachypodium hybridum*

**DOI:** 10.1111/tpj.14869

**Published:** 2020-07-03

**Authors:** Natalia Borowska‐Zuchowska, Ales Kovarik, Ewa Robaszkiewicz, Metin Tuna, Gulsemin Savas Tuna, Sean Gordon, John P. Vogel, Robert Hasterok

**Affiliations:** ^1^ Plant Cytogenetics and Molecular Biology Group, Institute of Biology, Biotechnology and Environmental Protection Faculty of Natural Sciences University of Silesia in Katowice Jagiellonska 28 Katowice 40‐032 Poland; ^2^ Department of Molecular Epigenetics Institute of Biophysics Academy of Sciences of the Czech Republic, v.v.i. Královopolská 135 Brno 612 65 Czech Republic; ^3^ Department of Field Crops Faculty of Agriculture Tekirdag Namik Kemal University Suleymanpasa Tekirdag 59030 Turkey; ^4^ Tekirdag Anatolian High School Suleymanpasa Tekirdag 59030 Turkey; ^5^ US Department of Energy (DOE) Joint Genome Institute (JGI) Berkeley CA 94720 USA; ^6^ University California Berkeley, Berkeley CA 94720 USA

**Keywords:** 35S rDNA evolution, 35S rRNA gene expression, allopolyploidy, *Brachypodium hybridum*, nucleolar dominance

## Abstract

Nucleolar dominance (ND) consists of the reversible silencing of 35S/45S rDNA loci inherited from one of the ancestors of an allopolyploid. The molecular mechanisms by which one ancestral rDNA set is selected for silencing remain unclear. We applied a combination of molecular (Southern blot hybridization and reverse‐transcription cleaved amplified polymorphic sequence analysis), genomic (analysis of variants) and cytogenetic (fluorescence *in situ* hybridization) approaches to study the structure, expression and epigenetic landscape of 35S rDNA in an allotetraploid grass that exhibits ND, *Brachypodium hybridum* (genome composition DDSS), and its putative progenitors, *Brachypodium distachyon* (DD) and *Brachypodium stacei* (SS). In progenitor genomes, *B*.* stacei* showed a higher intragenomic heterogeneity of rDNA compared with *B*.* distachyon*. In all studied accessions of *B*.* hybridum*, there was a reduction in the copy number of S homoeologues, which was accompanied by their inactive transcriptional status. The involvement of DNA methylation in CG and CHG contexts in the silencing of the S‐genome rDNA loci was revealed. In the *B*.* hybridum* allotetraploid, ND is stabilized towards the D‐genome units, irrespective of the polyphyletic origin of the species, and does not seem to be influenced by homoeologous 35S rDNA ratios and developmental stage.

## Significance Statement

Nucleolar dominance (ND) can be present in both hybrids and allopolyploids, and consists of the reversible selective suppression of the 35S rRNA genes that are inherited from one of the ancestral species. We showed that in the allotetraploid *Brachypodium hybridum* ND is stabilized towards the *Brachypodium distachyon*‐like rDNA units, irrespectively of species polyphyletic origin and genetic changes influencing homoeologue 35S rRNA gene ratios.

## INTRODUCTION

Allopolyploidy consists of interspecific hybridization followed by chromosome doubling, and has long been considered to be a major driving force in evolution (Liu and Wendel, 2003). However, the merger of two or more divergent genomes within one nucleus presents challenges to long‐term viability including: deletions, translocations, transposon activation and meiotic irregularities. In addition to these structural changes, the expression of the duplicated genes can result in the silencing of one of the homoeologues (Leitch and Leitch, 2008; Weiss‐Schneeweiss *et al*., 2013; Wendel *et al*., 2018). One example of an interaction between the ancestral genomes in allopolyploids that results in altered gene expression is nucleolar dominance (ND). ND was discovered in interspecific hybrids of *Crepis* (Asteraceae), in which the secondary constrictions were only carried by the chromosomes that had been inherited from one of the progenitors (Navashin, 1934). More than 30 years later, it was shown that the tandemly arranged 35S/45 S ribosomal DNA (rDNA) units encoding the 18S, 5.8S and 25–28S rRNAs are located within the secondary constrictions (Wallace and Birnstiel, 1966; Phillips *et al*., 1971). It was found that rDNA arrays may occupy one or more loci in a genome in which individual repeats do not evolve independently but evolve in a concerted manner (Kovarik *et al*., 2008; Garcia *et al*., 2017). Interestingly, the non‐coding subregions of the rDNA units, the internal transcribed spacers (ITS1 and ITS2) and the intergenic spacer (IGS) evolve faster than the coding sequences, and therefore are significantly more variable (Eickbush and Eickbush, 2007). Although there are hundreds to thousands of rDNA units per genome, not all rRNA genes undergo transcription. The excess of rDNA units bears epigenetic modifications that are characteristic of heterochromatin and that stay transcriptionally silent (Bockor *et al*., 2014; Costa‐Nunes *et al*., 2014).

The ND phenomenon consists of the reversible silencing of the 35S/45S rDNA loci that are inherited from one of the ancestral species, and has been described in allopolyploids and hybrids that belong to numerous and diverse plant genera (Pikaard, 2000). Although our understanding of the involvement of epigenetic modifications such as DNA methylation and histone deacetylation in establishing and maintaining the ND has advanced significantly in the last decades (Neves *et al*., 1995; Chen and Pikaard, 1997a; Lawrence *et al*., 2004; Earley *et al*., 2006; Costa‐Nunes *et al*., 2010), the mechanisms by which one ancestral rDNA set is selected for silencing remain elusive (Symonova, 2019). The proposed explanations that the uniparental expression of 35S rDNA corresponds to the physical characteristics of its IGSs (the so‐called enhancer imbalance hypothesis, originally proposed for *Xenopus* hybrids), or to differences in the affinity for transcription factors, are not supported in plants (Chen and Pikaard, 1997b; Frieman *et al*., 1999). It was also shown that ND in plant allopolyploids is not a maternal effect (Chen and Pikaard, 1997b). In *Arabidopsis thaliana* and *Hordeum vulgare*, the 35S rRNA genes were silenced based on the chromosomal position in which they resided (Nicoloff *et al*., 1979; Schubert and Künzel, 1990; Mohannath *et al*., 2016), and therefore the influence of the neighbouring DNA sequences on the rRNA gene expression cannot be ruled out.

Most of the research on ND has been performed on dicot species (e.g. *Arabidopsis suecica*, *Brassica* and *Nicotiana* allopolyploids), but rather less is known about this phenomenon in monocots, where the studies have been limited to *Triticum aestivum* (wheat; Guo and Han, 2014), which harbours very complex genomes with many rDNA loci. To fill a gap in the knowledge about ND in monocots we analysed its molecular basis in a small‐ and simple‐genome allotetraploid grass that exhibits ND, *Brachypodium hybridum* (2n = 30; genome composition DDSS). Its genomes most likely originated from two diploid progenitors: *B. distachyon* (2n = 10; DD) and *B. stacei* (2n = 20; SS). One of the features that made *B*.* hybridum* an amenable model in studies of rDNA evolution and expression is the fact that it contains only one rDNA locus per ancestral genome. Moreover, the whole genomic sequence of both diploid progenitors and several *B*.* hybridum* genotypes is available for the scientific community (IBI, 2010, https://phytozome.jgi.doe.gov). The preferential silencing of the *B*.* stacei*‐like rDNA loci was documented in different tissues of *B*.* hybridum*, including both meristematic (Idziak and Hasterok, 2008) and differentiated (Borowska‐Zuchowska *et al*., 2016) root cells, prophase‐I meiocytes, microspores and different tissues of immature and imbibed embryos (Borowska‐Zuchowska *et al*., 2019). As yet, there have been no reports on the expression status of the S‐genome rRNA genes in *B*.* hybridum* leaves. We have previously used molecular cytogenetic approaches to study the epigenetic status of 35S rDNA loci in *B*.* hybridum*. The actively transcribed D‐genome 35S rDNA units exhibited a low DNA methylation level and were enriched in euchromatic marks, such as acetylated histone isoforms (H4K5ac, H4K16ac and H3K9ac) (Borowska‐Zuchowska and Hasterok, 2017). In contrast, the *B*.* stacei‐*like rRNA genes were characterized by a high level of DNA methylation and were enriched with a heterochromatic marker: the dimethylated lysine 9 of histone H3 (H3K9me2). Interestingly, the global cytosine hypomethylation of the *B*.* hybridum* genome induced by 5‐azacytidine did not lead to a reactivation of the S‐genome rDNA loci, which strongly suggests the involvement of other factors that lie behind ND in this species (Borowska‐Zuchowska and Hasterok, 2017).

In order to shed more light on the molecular mechanisms that lie behind ND, we applied a combination of molecular and cytogenetic approaches to study the organization, expression and epigenetic landscape of 35S rDNA in 16 *B*.* hybridum* genotypes. The following questions were posed: (i) what is the level of the intragenomic homogeneity of the 35S rDNA units in the putative ancestors of *B*.* hybridum*; (ii) are the homoeologous 35S rDNA loci in different natural populations of *B*.* hybridum* faithfully inherited; (iii) does the expression status of the rRNA genes depend on their dosage in different accessions; and (iv) is DNA methylation involved in the preferential suppression of the S‐genome rDNA units?

## RESULTS

### Assembly of the D‐ and S‐genome 35S rDNA units, their sequence polymorphism and copy number

**Figure 1:**
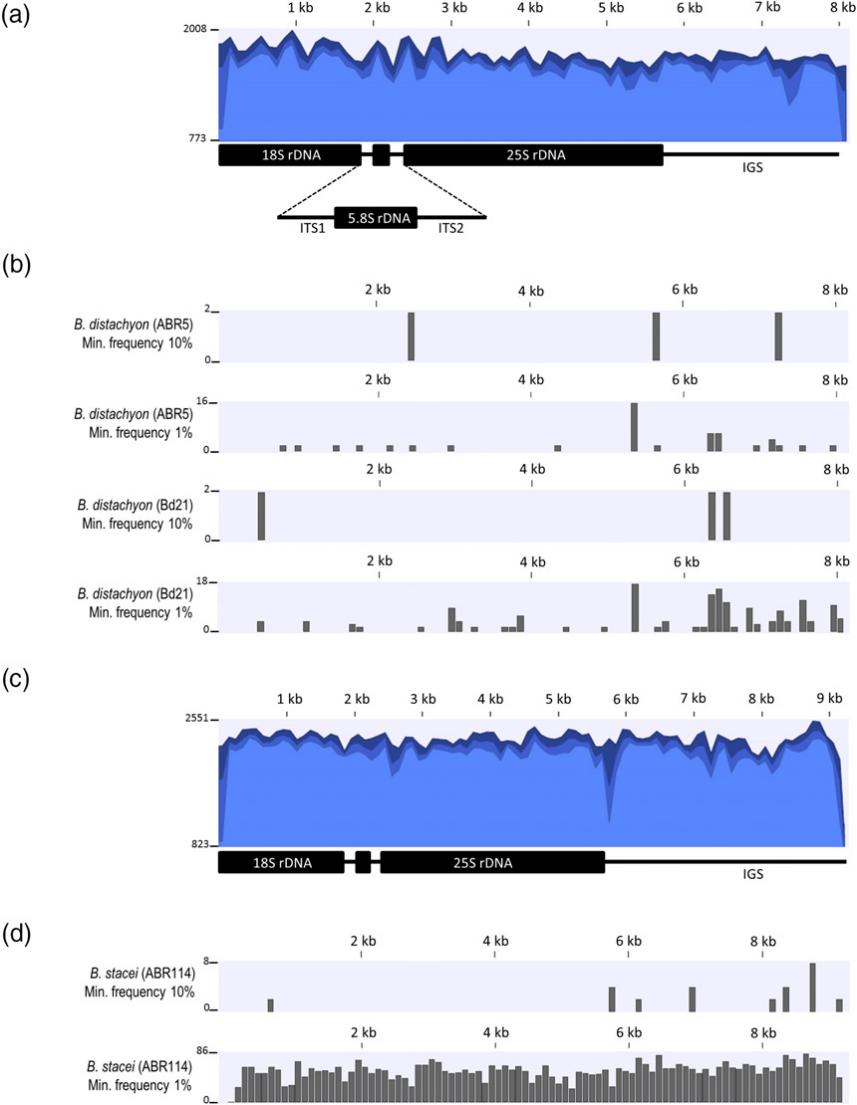
Intragenomic heterogeneity of the 35S rDNA units in *Brachypodium distachyon* (genotypes ABR5 and Bd21) and *Brachypodium stacei* (genotype ABR114) that were determined from the whole‐genomic Illumina reads. (a,c) A graphical display of the raw Illumina read coverage after mapping to the reference sequences: 35S rDNA consensus of *B. distachyon* (a) and *B. stacei* (c). The height of the graphs reflects the coverage of a specific region on the mapping. The three shades of blue represent the minimum, average and maximum coverage values for the aggregated mapped reads. (b,d) The distribution of the single‐nucleotide polymorphisms (SNPs) of high (minimum frequency 10%) and low (minimum frequency 1%) frequency in the 35S rDNA units of *B. distachyon* (b) and *B. stacei* (d). Each column on the graphs represents one or more SNP variants per 100 bp. The maximum number of SNPs per column is given on the left of each graph.

Although the complete genomic sequence of *B*.* distachyon* has been available since 2010 (IBI, 2010), the 35S rDNA unit has not yet been annotated. Based on the conserved nature of the 18S and 25S rRNA genes, we screened the genomic sequence of *B*.* distachyon* line Bd21 using the known homoeologous coding sequences of *Oryza sativa* (rice) as the queries. Two tandemly repeated sequences, located in the nucleolus‐organizing region (NOR)‐bearing chromosome Bd5, were identified. The 18S and 25S rDNA sequences of *B*.* distachyon* showed 98 and 96% nucleotide identity, respectively, with their counterparts from rice. The sequences of *B*.* distachyon* ITS1‐5.8S rDNA‐ITS2 as well as the sequence of the intergenic spacer (Bd IGS) have previously been published (Catalan *et al*., 2012; Borowska‐Zuchowska *et al*., 2016). These data allowed us to reconstruct the entire 35S rDNA unit that was used as the reference sequence for mapping the raw Illumina reads from two *B*.* distachyon* genotypes: Bd21 and ABR5. In both cases, the entire 35S rDNA unit, including the non‐coding sequences, was covered equally (Figure 1a). The read‐mapping details are provided in Table 1. A similar strategy was undertaken to reconstruct the complete 35S rDNA sequence from *B*.* stacei* by fusing raw genomic reads from the reference genotype ABR114. The entire 35S rDNA reference sequence was covered uniformly (Figure 1c; Table 1). From the mapped reads, we generated 35S rDNA consensus sequences for both diploids that were 8084‐bp‐ and 9215‐bp long for *B. distachyon* and *B. stacei*, respectively.

**Table 1:** Copy number of the 35S rDNA units in the *Brachypodium hybridum* allotetraploid and its putative ancestral species determined from the raw Illumina reads

Species	Genotype	Read archive accession	Mapped reads^a^	Total number of reads	GP^b^ [%]	GS^c^	Number of copies per 1C^d^
*Brachypodium distachyon*	Bd21	SRR4028751	46 295	11 888 585	0.39	1.20	665
	ABR5	SRR547946	32 478	11 969 038	0.27	0.84	463
*Brachypodium stacei*	ABR114	SRR3944698	28 826	10 710 800	0.27	0.74	410
*Brachypodium hybridum*	ABR113	SRR3945055	109 441	47 888 584	0.23	1.42	788

^a^ The length of 18S rDNA consensus sequence that was used as a reference was 1.81 kb.

^b^ The genome proportion (GP) was calculated from the number of mapped reads to the consensus sequence divided by the total number of reads in percentages.

^c^ The genome space (GS) was calculated as (genome size in Mb × GP)/100.

^d^ The copy number of the rDNA units was calculated as GS/size of a single 18S rDNA unit (0.00181 Mb). The following genome sizes were considered: *B. distachyon* 0.316 pg/1C ≈ 309 Mb; *B. stacei* 0.282 pg/1C ≈ 276 Mb; and *B. hybridum* 0.630 pg/1C ≈ 620 Mb (Catalan *et al*., 2012). The conversion of pg to Mb was made according to Dolezel *et al*. (2003).

The distribution of the single‐nucleotide polymorphisms (SNPs) in the 35S rDNA repeats of both putative ancestral species of *B*.* hybridum* was determined using the ‘Basic Variant Detector’ tool in the clc genomics workbench. We observed a slightly higher number of high‐frequency SNPs (≥10%) in the rDNA units of *B*.* stacei* than in either *B*.* distachyon* genotype (Figure 1b,d; Tables S1, S3, S5). As expected, the non‐coding IGS regions appeared to be more variable than the coding sequences, especially in the *B*.* stacei* rDNA units (Figure 1d). When the low‐frequency SNPs were also considered (decreasing a SNP call threshold to ≥1%), we found a threefold higher intragenomic variability in the *B*.* distachyon* Bd21 than in ABR5 (Figure 1b; Tables S2 and S4). Most of the SNPs, however, were located in the non‐coding regions. Interestingly, we found more than 3000 low‐frequency SNPs (Table S6) in *B*.* stacei* that were uniformly distributed across the entire 35S rDNA unit (Figure 1d) which did not accumulate in specific subregions. Substitutions were far more abundant than the InDels (Table S6).

The number of 35S rDNA units in *B*.* distachyon* and *B*.* stacei* was estimated based on the slot‐blot hybridization with 18S rDNA as a probe (Figure S1). The intensity of the hybridization signals was comparable between the species, thus indicating little variation. Based on radioactivity measurements, Bd21 had the highest number of rDNA units per 1C (approx. 800 copies). The second *B*.* distachyon* genotype, ABR5, had approximately 700 copies per 1C, while *B*.* stacei* genotype ABR114 had approximately 650 copies per 1C. The 35S rRNA gene copy number in both diploids and *B*.* hybridum* ABR113 was also confirmed via the *in silico* analysis of all genomic reads (Table 1). *Brachypodium hybridum* ABR113 had more rDNA copies than any of the progenitor accessions. The values calculated based on the number of mapped reads to the 18S rDNA reference sequences were congruent with those that had been experimentally determined by slot‐blot hybridization.

### **35S rDNA homoeologue ratios among the** ****B. hybridum**** **genotypes**

**Figure 2:**
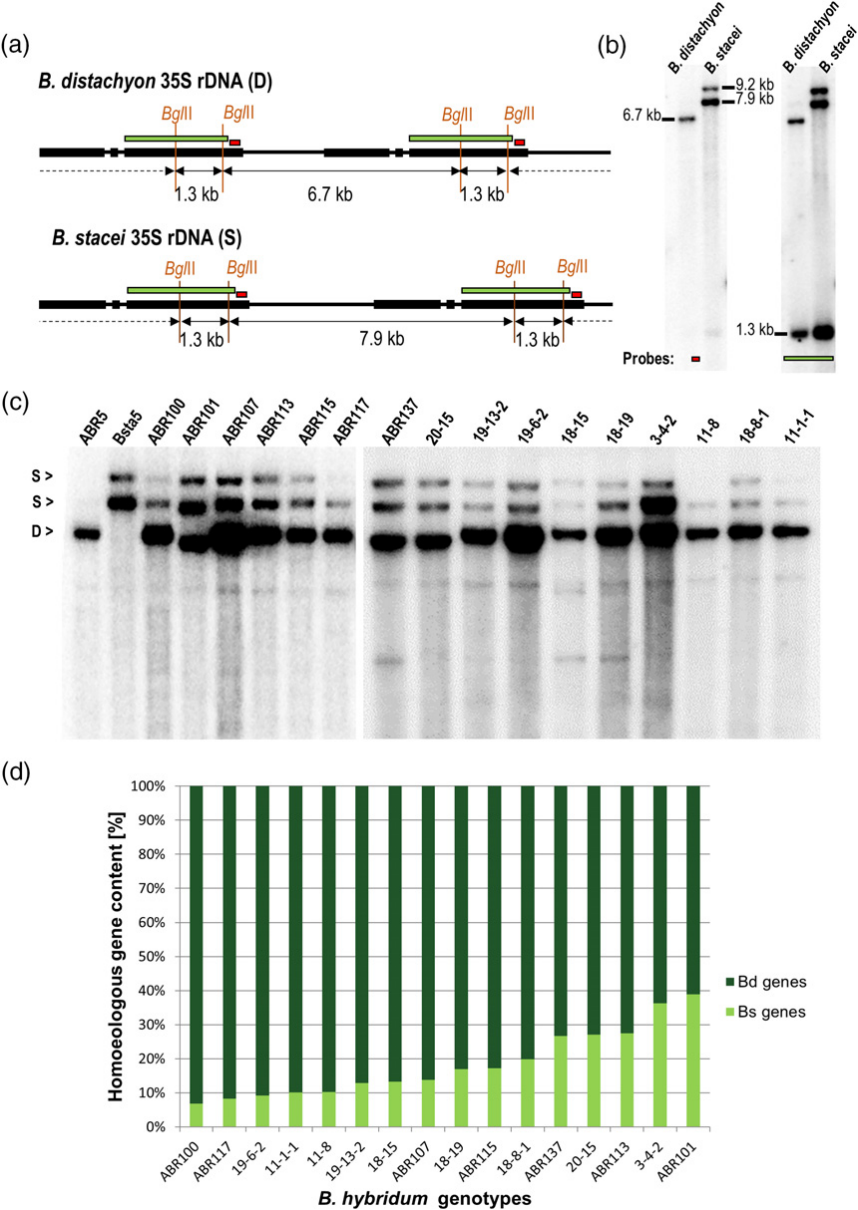
The structure of the 35S rDNA loci in *Brachypodium hybridum*. (a) The *Bgl*II restriction maps of the 35S rDNA units of *Brachypodium distachyon* and *Brachypodium stacei*. (b) Southern blot hybridization of the genomic DNA from *B. distachyon* and *B. stacei* that were subjected to digestion with *Bgl*II. The blot was hybridized with either the shorter (red rectangle, left panel) or longer (green rectangle, right panel) fragment of 25S rDNA. (c) Southern blot hybridization of the genomic DNA from *B*.* distachyon*, *B*.* stacei* and 16 *B*.* hybridum* genotypes that were subjected to digestion with *Bgl*II. The blot was hybridized with the short 25S rDNA probe. (d) Quantification of 35S rDNA homoeologues in different *B*.* hybridum* genotypes. The homoeologue gene number is denoted as a proportion of the D‐genome or S‐genome rDNA to the total rDNA.

In order to determine the ancestral 35S rDNA contributions, Southern blot hybridization with the 25S rDNA probe was performed on genomic DNA (gDNA) from 16 *B*.* hybridum* genotypes originating from diverse habitats and from two genotypes of each putative ancestral species, *B*.* distachyon* and *B*.* stacei* (Table 2). In the 35S rDNA consensus sequences of both diploid species, the two *Bgl*II restriction sites were found within the 25S rDNA coding sequence (Figure 2a). As was expected in *B*.* distachyon*, the short 25S rDNA probe hybridized to a single *Bgl*II fragment that was 6.7 kb long (Figure 2b, left panel). The long 25S rDNA probe variant hybridized to the 6.7‐ and 1.3‐kb fragments (Figure 2b, right panel), which was consistent with the restriction map (Figure 2a). Interestingly, in the case of both of the genotypes of *B*.* stacei*, the 25S rDNA probes hybridized to two and three *Bgl*II fragments for the short and long variants of the probe, respectively (Figure 2b). An additional, slow‐migrating band was consistent with the full‐size rDNA unit of *B*.* stacei*. Its presence may be explained by a mutation at one of the two *Bgl*II restriction sites. Indeed, a site located proximally to the 5.8S rRNA gene (site position: 3850–3855 nt) was less conserved than the distal one (site position: 5185–5190 nt), based on the higher number of SNPs according to the bioinformatic analysis (Table S6). The short 25S rDNA probe that had hybridized to one or two *Bgl*II fragments in *B*.* distachyon* and *B*.* stacei*, respectively (Figure 2b), was selected for Southern blot hybridization on the gDNA of *B*.* hybridum*. In all genotypes of the allotetraploid we observed rDNA variants that had derived from both ancestors, however, a reduction of *B*.* stacei* homoeologues was also denoted in some of the genotypes, e.g. in ABR100 (Figure 2c). To determine the contribution of the D‐ and S‐genome rDNA loci, the radioactivity of the hybridization bands was quantified using a phosphorimager. The D‐genome 35S rDNA units were more abundant than the S‐genome units for all of the studied genotypes. The contribution of the *B*.* stacei‐*inherited units varied from 7% in ABR100 to 39% in ABR101 (Figure 2d).

**Table 2:** General characteristics of the *Brachypodium* species used in this study

Species	Accession number	2n	x	Genome designation	Origin; coordinates	Source
*B. distachyon*	Bd21	10	5	DD	Iraq	A
	ABR5	10	5	DD	Spain, Huesca	B
*B. stacei*	Bsta5	20	10	SS	Spain, Alicante	C
	ABR114	20	10	SS	Spain, Formentera	B
*B. hybridum*	ABR100	30	5 + 10	DDSS	Iran, Khalaf Abad	B
	ABR101	30	5 + 10	DDSS	S. Africa, nr Darling, Cape	B
	ABR107	30	5 + 10	DDSS	Province, Greece	B
	ABR113	30	5 + 10	DDSS	Portugal, Lisbon	B
	ABR115	30	5 + 10	DDSS	S. Africa	B
	ABR117	30	5 + 10	DDSS	Afghanistan	B
	ABR137	30	5 + 10	DDSS	W. Australia	B
	3‐4‐2	30	5 + 10	DDSS	Turkey; 38°17′402″N, 27°24′139″E	D
	11‐1‐1	30	5 + 10	DDSS	Turkey; 36°32′086″N, 29°07′586″E	D
	11‐8	30	5 + 10	DDSS	Turkey; 36°32′086″N, 29°07′586″E	D
	18‐8‐1	30	5 + 10	DDSS	Turkey; 36°02′662″N, 32°40′504″E	D
	18‐15	30	5 + 10	DDSS	Turkey; 36°02′662″N, 32°40′504″E	D
	18‐19	30	5 + 10	DDSS	Turkey; 36°02′662″N, 32°40′504″E	D
	19‐6‐2	30	5 + 10	DDSS	Turkey; 36°10′641″N, 33°26′255″E	D
	19‐13‐2	30	5 + 10	DDSS	Turkey; 36°10′641″N, 33°26′255″E	D
	20‐15	30	5 + 10	DDSS	Turkey; 36°57′277″N 34°45′042″E	D

A, US Department of Agriculture, National Plant Germplasm System, Beltsville, MD, USA; B, Institute of Biological, Environmental and Rural Sciences, Aberystwyth University, Aberystwyth, UK; C, High Polytechnic School of Huesca, University of Zaragoza, Huesca, Spain; D, University of Silesia, Katowice, Poland.

**Figure 3:**
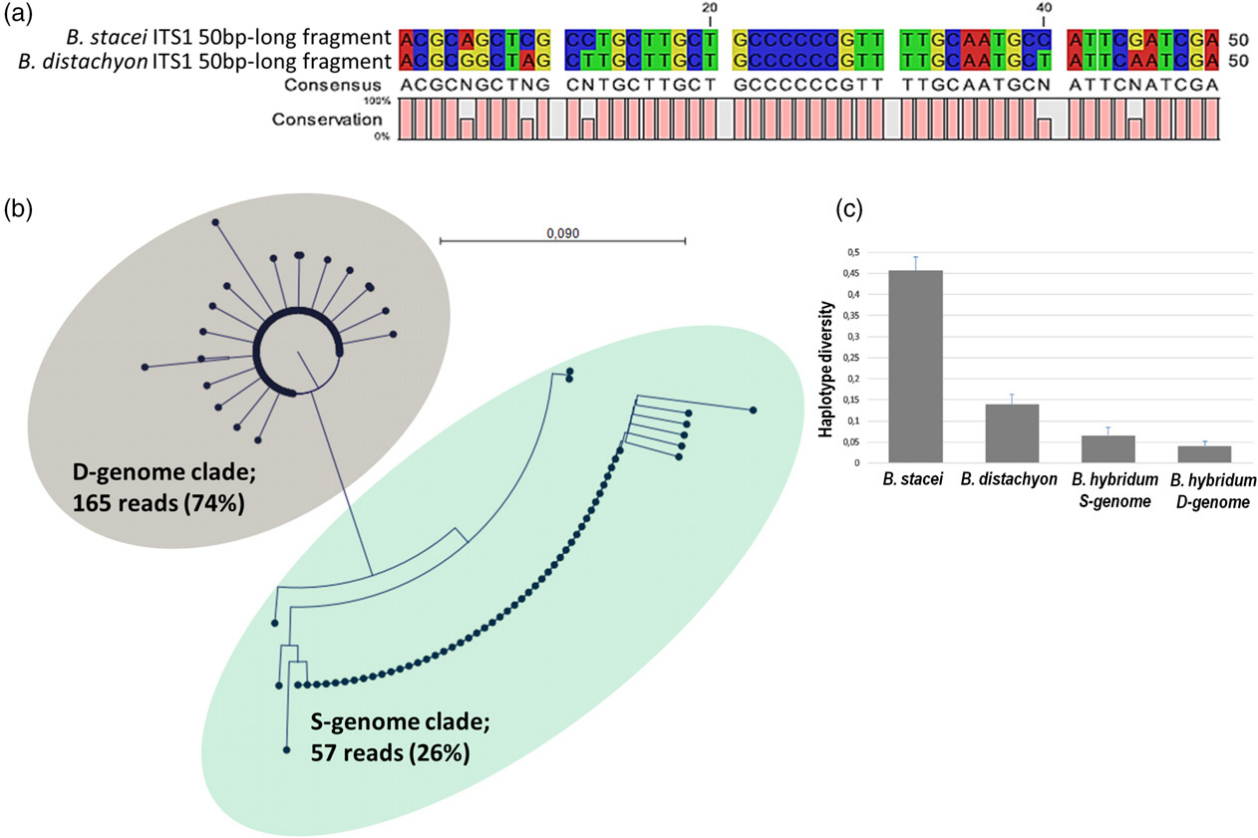
The D‐ and S‐genome 35S rDNA contributions in the *Brachypodium hybridum* ABR113 reference genotype. (a) Alignment of a 50‐bp ITS1 fragment from *B*.* distachyon* and *B*.* stacei*. (b) Neighbour‐joining phylogenetic tree constructed from 222 aligned Illumina reads, which were extracted from *B*.* hybridum* ITS1. (c) Haplotype diversity of ITS1 families in *B. hybridum* ABR113 subgenomes and its putative progenitor accessions. Note, the high intragenomic diversity of *B. stacei* ITS1 consistent with high number of SNPs in rDNA units (Figure 1).

Using raw reads from the genome of the *B*.* hybridum* reference genotype ABR113, we were able to determine *in silico* the ancestral rDNA contributions in this allotetraploid. We selected a 50‐bp‐long sequence that had derived from a variable part of the ITS1 subregion which had about a 10% divergence between the D‐ and S‐genome rDNA consensus sequences (Figure 3a). The S‐genome ITS1 fragment was BLASTed against the *B*.* hybridum* whole‐genome sequence library to identify 3706 sequences, which were then extracted, trimmed, sampled and aligned, as was described in the Experimental procedures. A neighbour‐joining phylogenetic tree was constructed based on the alignment of 222 reads (Figure 3b; Figure S2). The tree was composed of two branches: the first was highly homogenous and consisted of 165 (74%) D‐genome sequences and the second was composed of 57 (26%) more divergent S‐genome ITS1 sequences. On the Southern blot, phosphorimaging of homoeologous bands revealed 74% of the D‐genome rDNA and 26% of the S‐genome rDNA in ABR113 rDNA (Figure 2d). Thus, the rDNA ratios determined by *in silico* calculations were congruent with those of the Southern blot. The haplotypic diversity of S‐ and D‐genome ITS1 homoeologues was determined in S and D genomes of *B*.* hybridum* and its putative progenitors (Figure 3c). There was no significant difference (Pearson chi‐square test, *P* = 0.928) between both S‐ and D‐genome homoeologous families of *B*.* hybridum* ABR113. However, both *B*.* hybridum* families and those of *B*.* distachyon* had significantly lower (*P* < 0.001) haplotypic diversity than the *B*.* stacei* ITS1.

### **Number and localization of rDNA loci in the** ****B. hybridum**** **metaphase chromosomes and interphase nuclei**

**Figure 4:**
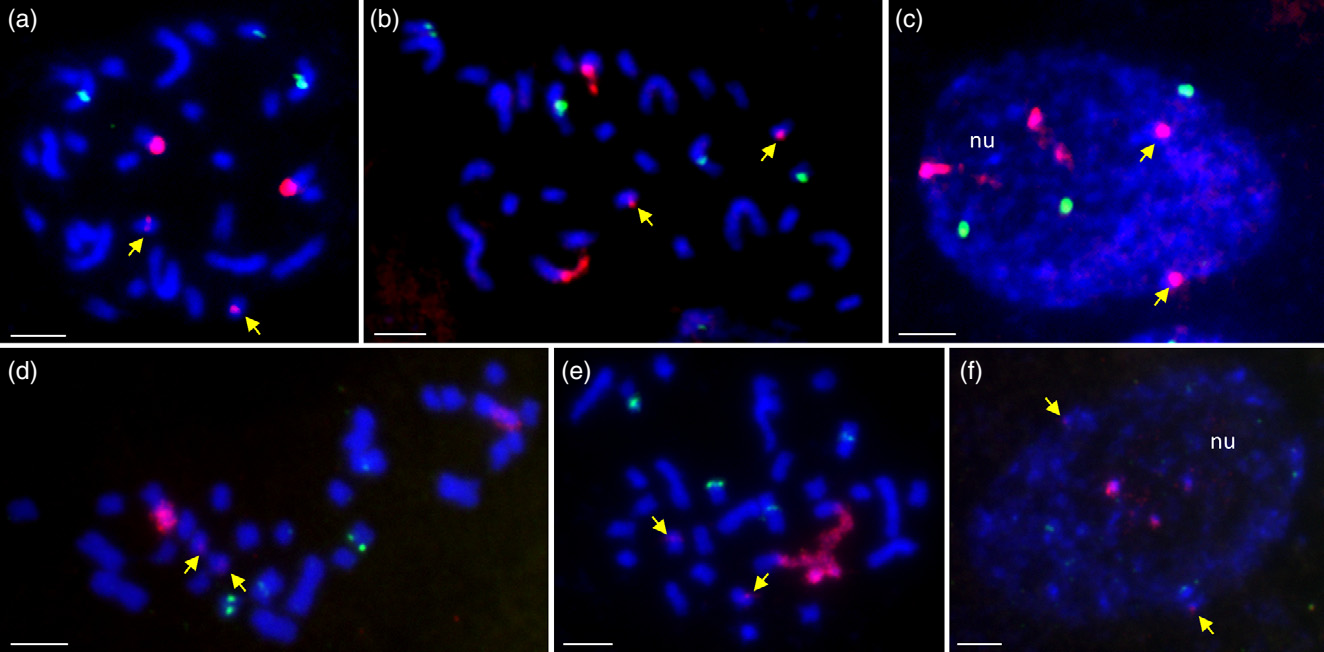
The distribution of the 35S and 5S rDNA loci in the mitotic metaphase chromosomes and interphase nuclei of *Brachypodium hybridum*. FISH with 25S rDNA (red fluorescence) and 5S rDNA (green fluorescence) as probes on the mitotic metaphase chromosomes (a,d), prometaphase chromosomes with visible secondary constrictions (b,e) and interphase nuclei (c,f) of the *B. hybridum* genotypes that showed the highest (ABR101, a–c) and the lowest (ABR100, d–f) S‐genome 35S rDNA content. The S‐genome 35S rDNA loci are indicated by yellow arrows. nu, nucleolus. Scale bars: 5 µm.

The number and localization of 35S and 5S rDNA loci were determined on the mitotic metaphase chromosomes from root‐tip cells of 14 *B*.* hybridum* genotypes using fluorescence *in situ* hybridization (FISH; data on genotypes ABR113 and ABR117 have already been published; Hasterok *et al*., 2004; Borowska‐Zuchowska *et al*., 2016). Simultaneous FISH using 25S rDNA and 5S rDNA probes identified two pairs of chromosomes bearing the 35S rDNA loci and another two chromosomal pairs that carried the 5S rDNA loci in all of the studied accessions (Figures 4 and S3–S16). Interestingly, the intensity of 25S rDNA hybridization signals was not uniform across the chromosomes: the signals in the chromosomes that had originated from *B*.* stacei* were smaller than those inherited from *B*.* distachyon*, especially in the genotype ABR100 (Figures 4d–f and S4). This accession had also the lowest contribution of S‐genome rDNA, revealed by the Southern blot hybridization (Figure 2c,d). We also found that only the terminally located 35S rDNA loci that had originated from the D‐genome were able to form secondary constrictions (Figure 4b,e; Figures S3–S16), while the proximally located S‐genome rDNA loci always remained condensed (Figures 4b,e and S2–S4). At the level of the interphase nuclei, the FISH signals that corresponded with the 25S rDNA that had been inherited from *B*.* distachyon* tended to be located around the nucleolus (Figures 4c,f and S3–S16), suggesting their transcriptional activity. In contrast, the *B*.* stacei*‐like 35S rDNA loci were located outside of the nucleolus (Figures 4c,f and S3–S16).

Additional FISH analysis with 25S rDNA as a probe was performed on nuclei isolated from leaves of *B*.* hybridum* ABR113 genotype. We found that only D‐genome 35S rDNA loci were located adjacent to the nucleolus, while the *B*.* stacei‐*derived ones were located in the nuclear periphery (Figure S17).

### **35S rDNA homoeologue expression in** ****B. hybridum****

**Figure 5:**
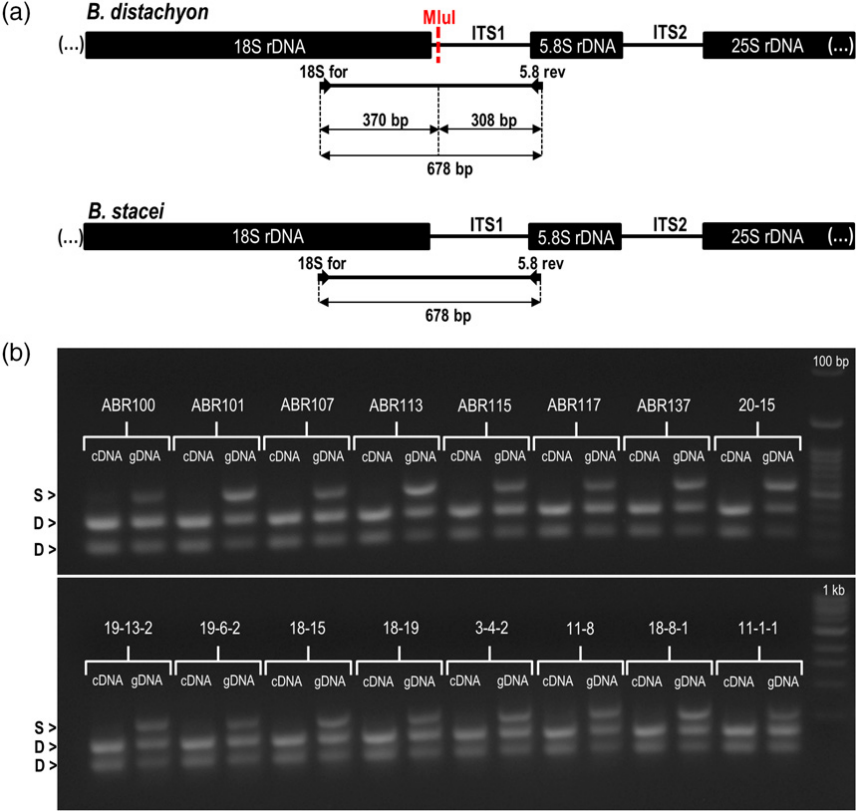
Expression analysis of the 35S rRNA genes in the 16 *Brachypodium hybridum* genotypes using the RT‐PCR CAPS method. (a) The *Mlu*I restriction profile of the S‐genome and D‐genome ITS1 amplification products and the expected sizes of the bands after digestion. (b) The gel restriction profiles of the ITS1 PCR products that were obtained from the leaf cDNA and gDNA from the 16 *B. hybridum* genotypes. Arrowheads denote the bands that corresponded with either S‐genome 35S rDNA (S) or D‐genome 35S rDNA (D).

To study the expression of the D‐ and S‐genome 35S rDNA in *B*.* hybridum* we analysed the rRNA precursors, taking advantage of the ITS1 sequence divergence. A bioinformatic analysis of the ITS1 sequences from both putative ancestral species revealed the presence of a single restriction site for *Mlu*I in the ITS1 of *B*.* distachyon*, while there was no site for this enzyme in the corresponding sequence of *B*.* stacei* (Figure 5a), which made their restriction patterns species‐specific. Using the reverse‐transcription cleaved amplified polymorphic sequence (RT‐CAPS) approach, we analysed the expression of rDNA homoeologues in leaf samples from 16 *B*.* hybridum* genotypes (Figure 5b). We detected both ancestral rDNA variants, evidenced by three bands in the ‘gDNA’ lanes. However, at the level of RNA, we only observed the *B*.* distachyon*‐inherited *Mlu*I fragments (two bands for ‘cDNA’ lanes), which implied a strong uniparental expression of the rRNA genes in the leaves of *B*.* hybridum*.

### **DNA methylation analysis of 35S rDNA homoeologues in** ****B. hybridum****

DNA methylation patterns of the rDNA units in the 16 genotypes of *B*.* hybridum* and in both of the putative ancestral species were determined using a Southern blot on gDNAs that had been digested with the methylation‐sensitive enzymes *Pst*I and *Xho*I, which recognize the methylated DNA sites in the CHG and CG sequence contexts, respectively. This provided a general outlook on the global differences in the cytosine methylation levels between the rDNA in *B*.* hybridum* that had derived from both progenitors.

**Figure 6:**
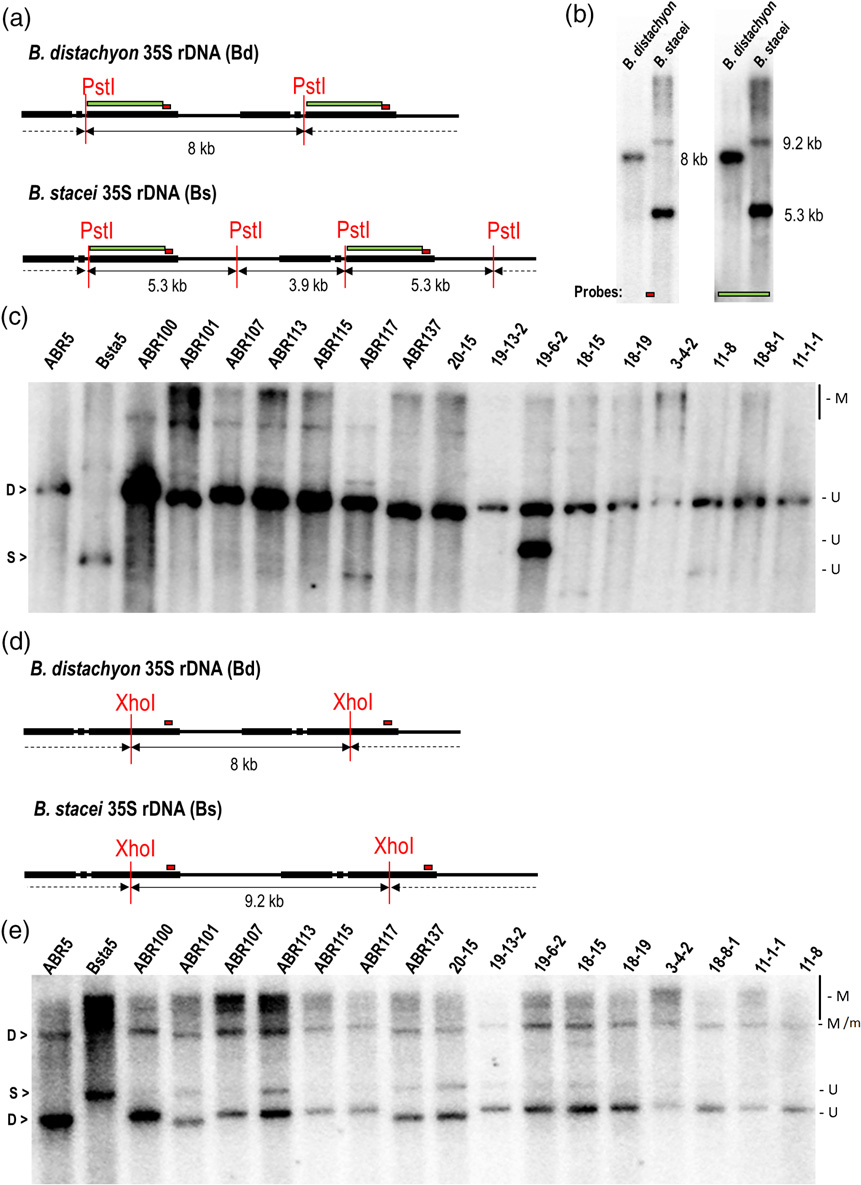
DNA methylation analysis of the 35S rDNA homoeologues in *Brachypodium hybridum*. (a,d) The *Pst*I (a) and *Xho*I (d) restriction maps of the 35S rDNA units of *Brachypodium distachyon* and *Brachypodium stacei*. (b) Southern blot hybridization of the genomic DNA from *B. distachyon* and *B. stacei* that had been subjected to *Pst*I digestion. The blot was hybridized with either a short (red rectangle, left panel) or long (green rectangle, right panel) fragment of 25S rDNA. (c,e) Southern blot hybridization of the genomic DNA from *B. distachyon*, *B. stacei* and the 16 *B. hybridum* genotypes digested with *Pst*I (c) or *Xho*I (e). The membranes that are presented on (c) and (e) were hybridized with a short 25S rDNA probe. M, methylated fragments; M/m, fragments resulting from the methylated or mutated sites; U, unmethylated fragments.

In the 35S rDNA consensus sequence of *B*.* distachyon*, a single *Pst*I restriction site was present in the ITS2 subregion (Figure 6a). As expected, both the short and long 25S rDNA probes hybridized to a single 8.0‐kb *Pst*I fragment, which reflected the full‐size non‐methylated rDNA unit of the D‐genome (Figure 6b). Two restriction sites for *Pst*I located in the ITS2 and IGS subregions were present in the consensus rDNA sequence of the second ancestor (Figure 6a). Both the short and long 25S rDNA probes hybridized to two *Pst*I fragments: the 5.3‐ and 9.2‐kb bands (Figure 6b), thus indicating non‐methylated units. The presence of an additional, slow‐migrating fragment that reflected a full‐size rDNA unit may be explained by either a mutation or the methylation of cytosine in one of the two *Pst*I restriction sites. Interestingly, in the case of the *B*.* stacei* genotypes, a large amount of high‐molecular‐weight DNA hybridized to the 25S rDNA (Figure 6b). In all 16 *B*.* hybridum* accessions, a strong D‐genome rDNA band of 8.0 kb was observed, indicating the absence of D‐unit methylation at the *Pst*I site. In contrast, with the exception of the ABR117 genotype, no demethylated 5.3‐kb band was visualized in *B*.* hybridum*, while there was a 9.2‐kb partially methylated band and a smear of high ‐molecular ‐weight (>10 kb) heavily methylated fragments. Thus the 35S rDNA sequences that originated from the *B*.* stacei* ancestor were almost completely methylated at *Pst*I sites in *B*.* hybridum*. An additional strong band migrating in the 5‐kb region (marked as ‘u’, not present in any of the ancestors) was visualized in the 19‐6‐2 accession (Figure 6c) and may reflect a genetic variation between the progenitors.

The bioinformatic analysis revealed the presence of a single *Xho*I restriction site in the 35S rDNA consensus sequences from the putative progenitor species of *B*.* hybridum* (Figure 6d). In *B*.* distachyon* ABR5, the 25S rDNA probe hybridized to two *Xho*I fragments: the fast‐migrating band, which was 8‐kb long, and a larger fragment that reflected two rDNA units, in one of which a cytosine was methylated (Figure 6e). In the *B*.* stacei* Bsta5 line, a single *Xho*I fragment (9.2 kb) and a smear of unresolved high‐molecular‐weight fragments were observed in the upper part of the membrane (Figure 6e). The *Xho*I fragments of *B*.* distachyon* origin were present in all of the *B*.* hybridum* accessions, indicating the absence of CG methylation in these homoeologues (Figure 6e). The unmethylated 9.2‐kb *Xho*I fragment of *B*.* stacei* origin was barely visible in the ABR101, ABR113, ABR137, 20–15 and 18–15 *B*.* hybridum* genotypes. In contrast, a smear of high‐molecular‐weight methylated fragments inherited from the *B*.* stacei* parent was clearly visible. These results indicate that the methylation level of *B*.* distachyon* homoeologues remained unchanged in a hybrid while those of *B*.* stacei* became hypermethylated.

## DISCUSSION

### **Sequence polymorphism of the 35S rDNA units in** ****B. distachyon**** **and** ****B. stacei****

The genes encoding for 18S‐5.8S‐25S ribosomal RNAs are known to evolve in a concerted manner. Unequal crossing over and gene conversion are believed to drive the homogenization of the rDNA units, and therefore their sequence homogeneity within an array is higher than would be expected by a random mutation (Szostak and Wu, 1980; Naidoo *et al*., 2013). Congruent with this hypothesis, we detected a relatively low intragenomic variability of 35S rDNA units, ranging from up to three polymorphic sites in *B*.* distachyon* and up to 14 polymorphic sites in *B*.* stacei* (for SNPs frequency ≥10%). As was expected, most of the variation was in the non‐coding ITS and IGS subregions, which are under weaker selection constraints than the coding rDNA sequences. Such a distribution pattern of SNPs in the rDNA units has also been observed in other organisms, e.g. *A*.* thaliana* (Rabanal *et al*., 2017), *Nicotiana* species (Lunerova *et al*., 2017), some mosses (Liu *et al*., 2013; Rosato *et al*., 2016) and some fungi (Ganley and Kobayashi, 2007). Interestingly, we detected a higher number of SNPs and increased ITS1 diversity in *B*.* stacei* rDNA than in *B*.* distachyon* rDNA (both genotypes). It is worth noting that SNPs were uniformly distributed in the coding‐ and non‐coding subregions. Such a mutation pattern could be explained by the presence of putative pseudogenized 35S rDNA copies in the *B*.* stacei* genome. In support, the mutations affecting the abundant *Cycas revoluta* (gymnosperm) rDNA pseudogenes occurred at nearly the same frequency in the coding and non‐coding subregions (Wang *et al*., 2016). Of note, the pseudogenized rDNA sequences were found in the human genome, mostly in the proximal regions (Robicheau *et al*., 2017). Similarly, the *B*.* stacei* rDNA loci are pericentromerically located (Borowska‐Zuchowska *et al*., 2016), suggesting that the propensity to pseudogenization may be increased in these chromosome areas. Certainly, more genomic studies are needed to support the hypothesis.

### **Variable inheritance of 35S rDNA homoeologues in the different** ****B. hybridum**** **accessions**

At least three different evolutionary scenarios have been observed for the 35S rDNA homoeologues in natural plant allopolyploids to date (Volkov *et al*., 2007): (i) the presence of rDNA homoeologues from both parents without any visible changes (Volkov *et al*., 1993); (ii) a uniparental rDNA inheritance as a result of the elimination/conversion of the rDNA units that had been inherited from the second parent (Wendel *et al*., 1995; Kotseruba *et al*., 2003; Dadejova *et al*., 2007; Lim *et al*., 2007; Volkov *et al*., 2017; Kolano *et al*., 2019); or (iii) a uniparental rDNA inheritance that is followed by rearrangements and the appearance of new rDNA classes (Clarkson *et al*., 2005; Kovarik *et al*., 2008). As is shown in the present work, the first scenario, i.e. genetic additivity, does not apply to the allotetraploid *B*.* hybridum*. Although the 35S rDNA units that had derived from both progenitors were present in all of the studied accessions of *B*.* hybridum*, a marked reduction in copy number of the S‐homoeologues occurred during their evolution (Figure 2). This trend was also confirmed in the reference genotype ABR113 using the available genomic approaches. The S‐genome rDNA contributions to total rDNA varied from 7 to 39% between different *B*.* hybridum* genotypes. This variation in the rDNA ratios most likely is related to the polyphyletic origin of *B*.* hybridum*. Recent dating analysis revealed that different *B*.* hybridum* lines arose from multiple crosses between *B*.* distachyon*‐ and *B*.* stacei*‐like ancestral species during the Quaternary, ca. 0.05–1 Ma (Catalan *et al*., 2012; Diaz‐Perez *et al*., 2018), thus the elimination of the S‐genome rDNA may still be in progress. Interestingly, the haplotypic diversity of S‐genome units was markedly lower in *B*.* hybridum* compared to parental *B*.* stacei*, suggesting that the divergent and perhaps pseudogenized units were preferentially eliminated from the *B*.* hybridum* genome. Alternatively, rDNA homoeologues could still homogenize despite their inactivity. Considering the relatively ancient origin of *B*.* hybridum,* the homogenization of 35S rDNA proceeds relatively slower than in other allopolyploid systems of a comparable age (Dadejova *et al*., 2007; Kolano *et al*., 2019). Interestingly, there were no intergenomic translocations between the D and S genomes of the *B*.* hybridum* allotetraploid (Lusinska *et al*., 2018). It is therefore possible that the relatively slow tempo of rDNA homogenization may reflect an overall stasis of the *B*.* hybridum* subgenomes.

As previously shown for many allopolyploids, one parental set of rDNA can be overwritten by the rDNA units derived from the second parental species. Such a process of sequence conversion may encompass the whole parental rDNA set (e.g. in *Nicotiana arentsii*) or just a portion of the rDNA units that had derived from one progenitor (e.g. *Nicotiana rustica*, *Nicotiana tabacum*, *Brassica napus* and *Thinopyrum intermedium*; Kovarik *et al*., 2008; Mahelka *et al*., 2013; Sochorova *et al*., 2017). The complete homogenization towards the D‐genome rDNA units in *B*.* hybridum* may be excluded (at least in the accessions analysed by us), as we detected S‐rDNA variants in all of the accessions studied by Southern blot hybridization. Moreover, in the highly stringent FISH experiments, only the proximally located S‐genome rDNA loci were detected in the metaphase chromosomes of the* B*.* hybridum* ABR113 using a *B*.* stacei*‐specific IGS probe (Borowska‐Zuchowska *et al*., 2016). Although the partial homogenization of the S‐genome rDNA units cannot be ruled out, the size of the FISH hybridization signals that corresponded with 25S rDNA in the *B*.* stacei‐*inherited chromosomes seems to corroborate a reduction of the S‐genome rDNA units. For instance, in the ABR100 accession in which the contribution of the S‐units was 7% of the total 35S rDNA, we observed significantly weaker rDNA FISH signals in the *B*.* stacei*‐like chromosomes than in the *B*.* distachyon* ones.

Interestingly, the S‐genome 35S rDNA units were present in the ABR117 genotype of *B*.* hybridum*. The contribution of S‐genome 35S rDNA units to the total number of rDNA units was low (8%) in this accession, however, only one of the two S‐genome rDNA families that were visible after *Bgl*II restriction was detected in this genotype. Our previous analysis using FISH with 25S rDNA as a probe on the mitotic metaphase chromosomes of ABR117 revealed only the presence of terminally located D‐genome rDNA units, which suggests that the S‐genome repeats were completely eliminated (Hasterok *et al*., 2004; Borowska‐Zuchowska *et al*., 2016). It seems that both the reduction of the number of S units and the elimination of one S‐genome rDNA family prevented the visualization of these loci *in situ*.

In the case of the D‐genome 35S rDNA loci, length polymorphism was detected among different *B*.* hybridum* genotypes. In the ABR101, ABR137 and 20–15 lines, slightly shorter restriction fragments were detected than in the parental *B*.* distachyon* rDNA. Such a polymorphism is most likely caused by the length variability in the intergenic spacers of 35S rRNA genes, as shown in other plant species, e.g. *Atropa*, *Nicotiana* and *Prunus* (Volkov *et al*., 1993; Borisjuk *et al*., 1997; Volkov *et al*., 2017). Since *B*.* hybridum* is a polyphyletic species we cannot exclude the possibility that these IGS polymorphisms were already present among the populations of progenitor species that donated their rDNAs to the allotetraploid.

### **Nucleolar dominance of the D‐genome rDNA in** ****B. hybridum**** **leaves and root tips**

As has been shown in many allopolyploids, because the repressed rDNA loci may be reactivated at certain developmental stages, ND seems to be a reversible, developmentally modulated phenomenon (Volkov *et al*., 2007). For instance, in synthetic *Solanum* allopolyploids, the reactivation of the silenced 35S rRNA genes was observed in the callus and anthers (Komarova *et al*., 2004). Similarly, in the allotetraploid *Brassica napus*, *Brassica oleracea*‐derived rRNA genes were activated after the floral meristem transition. Interestingly, transcripts of *Brassica oleracea* origin were found in all floral organs of *Brassica napus*, i.e. the floral buds, petals, sepals, anthers and siliques (Chen and Pikaard, 1997b). However, the studies of Sochorova *et al*. (2017) on different *Brassica napus* cultivars showed only a weak expression of *Brassica oleracea‐*inherited rDNA in the flower buds of only a few of accessions that were studied, thus the developmental regulation of ND may even be genotype specific. This fact is further supported by the *Brassica napus* cultivar ‘Norin 9’, in which a co‐dominance of *Brassica rapa*‐ and *Brassica oleracea*‐inherited 35S rDNA was present in the leaves, roots and flower buds (Sochorova *et al*., 2017). A co‐dominant expression profile of the parental rDNA in *Brassica napus* was also observed in the meristematic cells of 2‐ and 3‐day‐old roots (Hasterok and Maluszynska, 2000). In *Arabidopsis suecica*, which is another dicot allotetraploid that exhibits ND, a gradual silencing of the *A*.* thaliana*‐derived 35S rDNA loci occurred during early postembryonic development (Pontes *et al*., 2007). In contrast, in wheat–rye hybrids, it was found that the ND may be fully established as early as 4–5 days after fertilization (Castilho *et al*., 1995), thus ND regulation may vary significantly between allopolyploid species.

To date, D‐ and S‐genome rDNA activity was studied mainly in the reference genotype of *B*.* hybridum*, ABR113. It was shown that the *B*.* distachyon*‐inherited 35S rRNA genes are dominant at all developmental stages studied, including vegetative (Borowska‐Zuchowska *et al*., 2016; Borowska‐Zuchowska and Hasterok, 2017) and generative tissues (Borowska‐Zuchowska *et al*., 2019). Unlike in diploid allopolyploids, e.g. *Brassica napus* (Chen and Pikaard, 1997b), in *B*.* hybridum* the repressed S‐genome rRNA genes are not reactivated in generative tissues. Moreover, it was found that in contrast to developmentally unstable ND in Brassicaceae polyploids, ND is abolished neither in different tissues of immature embryos nor during early postembryonic development in *B*.* hybridum* (Borowska‐Zuchowska *et al*., 2019). In this work, we found that only D‐genome 35S rDNA loci are transcriptionally active in the leaf tissue of *B*.* hybridum*. We also revealed that the S‐genome loci were unable to form nucleoli in the interphase and correspondingly were not located within the secondary constrictions of the chromosomes in the root‐tip meristematic cells of any of the 16 accessions studied. Since the ability of the 35S rDNA loci to form a nucleolus is an indirect measure of their transcriptional activity (Shaw, 2013), we conclude that a strong uniparental expression of the D‐genome genes is present in both the leaf and root‐tip cells of all of the studied *B*.* hybridum* genotypes. Based on the data presented here, the S‐genome rDNA repression is not dependent on the ancestral rDNA ratios in the studied *B*.* hybridum* accessions.

### Involvement of DNA methylation in the suppression of S‐genome rDNA

Using two different enzymes that recognize methylated cytosines in the CHG and CG contexts, we found a differential DNA methylation status of the 35S rDNA homoeologues in *B*.* hybridum* (Figure 6). A large portion of the D‐genome 35S rDNA units was not methylated at the sites studied. The S‐genome bands that reflected the unmethylated rDNA loci from the second ancestral species, however, were either absent or significantly weaker than the D‐genome ones (Figure 6). These data are in agreement with studies on numerous plant hybrids and allopolyploids in which the involvement of DNA methylation in establishing and maintaining the ND was shown (Vieira *et al*., 1990; Houchins *et al*., 1997; Komarova *et al*., 2004; Lawrence *et al*., 2004; Costa‐Nunes *et al*., 2010; Dobesova *et al*., 2015). In the allotetraploid *A*.* suecica*, the small interfering RNA (siRNA)‐directed DNA methylation pathway is required to inactivate the 35S rDNA of an *A*.* thaliana* origin (Preuss *et al*., 2008). According to the proposed model of rRNA gene silencing, the siRNA molecules direct *de novo* DNA methyltransferase DRM2 activity to both the promoter and regulatory sequences that are present within the IGS of the targeted 35S rDNA unit. CHH methylation is then recognized by the methylcytosine‐binding protein MBD6, which is thought to react with other co‐repressors (Preuss *et al*., 2008; Costa‐Nunes *et al*., 2010). This crosstalk may mediate methylation in both the CG and CHG sequence contexts in the targeted rDNA units (Guo and Han, 2014). Thus, significant differences in the DNA methylation levels between the transcriptionally active and repressed rDNA units are observed in different plant species that exhibit ND (Neves *et al*., 1995; Houchins *et al*., 1997; Chen and Pikaard, 1997a; Komarova *et al*., 2004; Guo and Han, 2014).

Similar differences in the DNA methylation patterns between the 35S rDNA loci from the D and S genomes in *B*.* hybridum* were revealed using the molecular cytogenetics approaches (Borowska‐Zuchowska and Hasterok, 2017). The repressed S‐genome units were characterized by a significantly higher DNA methylation level than the D‐genome units, which is in agreement with the Southern blot analysis that is presented here (Figure 6). Interestingly, it was also revealed that the S‐genome rDNA units were not reactivated after the global hypomethylation of the *B*.* hybridum* genome induced by 5‐azacytidine (Borowska‐Zuchowska and Hasterok, 2017), which strongly suggests that DNA methylation alone is not sufficient to establish and maintain the ND in the studied allotetraploid. We speculate that the non‐methylated *B*.* stacei* units were methylated *de novo* in the allotetraploid as a result of epigenetic silencing and were shifted to the heterochromatic fraction.

To date, neither reverse dominance, i.e. the silencing of the D‐genome rDNA loci, nor co‐dominance of the D‐ and S‐genome rDNA have been reported in any tissue of *B*.* hybridum* (Idziak and Hasterok, 2008; Borowska‐Zuchowska *et al*., 2016; Borowska‐Zuchowska *et al*., 2019), which suggests that the S‐genome rDNA units may be irreversibly inactivated. However, investigations of the activity of the 35S rDNA homoeologues during early stages of embryo development in *B. hybridum*, as well as further analyses of the rDNA expression in different *B. hybridum* genotypes that have a more balanced ratio of parental rDNA copies, need to be performed in order to confirm our hypothesis. It is possible that strong silencing and methylation may be related to the proximal, pericentromeric position of the *B. stacei* NORs. In support of this, the ‘always’ dominant D‐genome NORs are located at the chromosome termini. There is some evidence that the chromosomal position of the rDNA locus may influence its activity (Schubert and Künzel, 1990; Neves *et al*., 2005; Báez *et al*., 2019). For instance, the 5S rDNA arrays that are located proximally to the centromeres are more methylated than the units that are located in distal positions in *A. thaliana* (Mathieu *et al*., 2002). The relationship between the chromosomal position of NOR, array homogeneity, chromatin condensation, epigenetic state and rDNA behaviour in allopolyploids requires further investigation.

## EXPERIMENTAL PROCEDURES

### Plant material

Twenty genotypes of three *Brachypodium* species, *B. hybridum*, *B. distachyon* and *B. stacei*, were used in this study. Information on their origins and basic cytogenetic characteristics are listed in Table 2. Nine *B. hybridum* genotypes were derived from the T_1_ generation of the germplasm that we had recently collected in climatically diverse habitats in Turkey (Table 2). The seeds were sown in pots with soil mixed with vermiculite (3∶1, w/w) and grown at 22°C and a 16‐h light/8‐h dark photoperiod in a greenhouse.

For the cytogenetic analyses, the seeds were grown on a filter paper moistened with tap water for 3 days at 20–22°C in the dark. Whole seedlings with approximately 2‐cm‐long roots were immersed in ice‐cold water for 24 h, fixed in 3:1 (v/v) methanol:glacial acetic acid at 4°C overnight and stored at −20 °C.

### ****In silico**** **reconstruction of the** ****B. distachyon**** **and** ****B. stacei**** **35S rDNA units, SNP calling and copy‐number estimation**

To annotate the *B. distachyon* and *B. stacei* 35S rDNA units, the following sequences were used as queries in the Bd21 and ABR114 genomic sequence screening:

- 18S rDNA sequence from rice (GenBank ID: X00755.1) (Takaiwa *et al*., 1984);
- 25S rDNA sequence from rice (GenBank ID: M11585.1) (Takaiwa *et al*., 1985);
- *B. distachyon* and *B. stacei* ITS1‐5.8S‐ITS2 sequences (GenBank IDs: JN187608.1 and JN187611) (Catalan *et al*., 2012); and
- *B. distachyon* and *B. stacei* IGSs (GenBank IDs: KX263276 and KX263278) (Borowska‐Zuchowska *et al*., 2016).

The sequences were ordered into 18S‐ITS1‐5.8S‐ITS2‐25S‐IGS rDNA units using clc genomics workbench (Qiagen, https://www.qiagen.com). The complete units were used as the reference sequences in the *B. distachyon* and *B. stacei* raw Illumina reads mapping using the ‘Map Read Reference’ tool. The following genomic libraries were used: *B. distachyon* Bd21 (SRP081838), *B. distachyon* ABR5 (SRP001538) and *B. stacei* ABR114 (SRP025051). All of the reads that contained any ambiguous nucleotides; were shorter than 100 bp (in the case of the Bd21 and ABR5 libraries) or were shorter than 150 bp (in the case of the ABR114 library); and/or failed to pass a quality score limit of *P* = 0.05 were removed using the ‘TRIM’ command in clc genomics workbench. The mapping parameters were as follows: mismatch cost, 2; insertion cost, 3; deletion cost, 3; with the length fraction set at 0.5 and the similarity fraction set at 0.8. The consensus 35S rDNA sequences were then extracted from the mapped reads.

For SNP calling, the ‘Basic Variant Detector’ tool in clc genomics workbench was used. The parameters were as follows: minimum read coverage, 300; minimum read count, 30; and minimum frequency (the ratio of ‘the number of countable reads supporting the allele’ to ‘the number of countable reads that covered the position of the variant’: ≥10% for high‐frequency SNPs or ≥1% for low‐frequency SNPs).

The copy number of the 35S rDNA units was calculated from the NGS read count using the following scheme: (i) the genome proportion (GP) was calculated from the number of mapped reads to the 18S rDNA consensus sequences divided by the total number of reads in percentages; (ii) the genome space (GS) of 35S rDNA was determined using the formula GP × size of the genome in Mb (309, 276 and 620 Mb for *B. distachyon*, *B. stacei* and *B. hybridum*, respectively); and (iii) the copy number of 35S rDNA was calculated as the GS value divided by the size of a 18S rDNA in Mb (0.00181 Mb).

### ****In silico**** **rDNA haplotypic analysis of the** ****B. hybridum**** **reference genotype**

A short 50‐bp‐long ITS1 fragment that showed a 10% sequence variation between the D‐ and S‐genome 35S rDNA consensus sequences was selected for the haplotypic analysis. The *B. stacei‐*like ITS1 fragment was BLASTed against a local whole‐genomic *B. hybridum* database (genotype ABR113, accession no. SRP025053). The hit sequences were trimmed for lengths (≥50 nt) and quality (no ambiguous nt were allowed; quality score limit *P* = 0.05), and then sampled for approximately 220 reads. The sampled reads were aligned using the ‘Multiple Alignment’ tool in clc genomics workbench with the following parameters: gap open cost, 10; gap extension cost, 1; end gap cost, as any other; and alignment mode, very accurate. A neighbour‐joining phylogenetic tree was constructed in clc genomics workbench using the Jukes–Cantor distance algorithm and a bootstrap of 100 replicates.

Intragenomic diversity was estimated by calculating the number of haplotypes in the aligned sequences. Each alignment contained 400 reads of each *B. stacei*, *B. distachyon* and *B. hybridum* ABR113_D‐genome ITS1, and 326 reads from *B. hybridum* ABR113_S‐genome ITS1. All sequences were of the same length (50 bp). DNA polymorphisms were calculated using a stand‐alone dnasp6 program (Rozas *et al*., 2017). Haplotype diversity was calculated as the number of haplotypes divided by number of sequences.

### DNA isolation and Southern blot hybridization

Total genomic DNA (gDNA) from the leaves from 1‐month‐old plants of diploid and allotetraploid *Brachypodium* species (Table 2) was isolated using a cetyltrimethylammonium bromide (CTAB) buffer as described by Doyle (1991). The gDNA was treated with 50 μg RNase for 1 h at 65°C. The samples were extracted with chloroform:isoamyl alcohol (24:1) and precipitated with isopropanol. The purified gDNAs (0.5–1 μg per sample) were digested with the restriction enzymes *Bgl*II, *Pst*I or *Xho*I, separated by gel electrophoresis on 1% (w/v) agarose gel and alkali‐blotted onto nylon membranes (Hybond XL; GE Healthcare Life Sciences, now Cytiva, https://www.cytivalifesciences.com), as described in Kovarik *et al*. (2005). *Bgl*II cuts the AGATCT motif and is methylation insensitive. *Xho*I cuts the CTCGAG motif and is sensitive to the methylation of the second C, while *Pst*I cuts the CTGCAG motif and the cleavage is blocked by the methylation of either C. The membranes were hybridized with a ^32^P‐labelled DNA probes (DekaLabel kit; ThermoFisher Scientific, https://www.thermofisher.com) using either the approximately 220‐bp PCR product derived from the 3′ end of the 25S rDNA of *Nicotiana tabacum* (tobacco; short probe; red rectangle in Figure 2a; forward primer, 5′‐GAATTCACCAAGTGTTGGAT‐3′; reverse primer, 5′‐AGAGGCGTTCAGTCATAATC‐3′) or a long 25S rDNA probe of approximately 2470 bp (long probe; green rectangle in Figure 2a) that contained an *Eco*RI‐*Eco*RI fragment of the 25S rRNA gene from *Solanum lycopersicum* (tomato) (Kiss *et al*., 1988). The Southern hybridization followed the procedure described in Kovarik *et al*. (2005). The hybridization bands were visualized with a phosphorimager (Typhoon 9410; GE Healthcare, https://www.gehealthcare.com) and the radioactivity of the bands was quantified with imagequant (GE Healthcare). At least two individuals from each *B. hybridum* population were analysed.

The slot‐blot hybridization with a 1.7‐kb fragment of the tobacco 18S rDNA as a probe was performed with 200, 100 and 50 ng of the genomic DNA of *B. distachyon* (genotypes Bd21 and ABR5) and *B. stacei* (genotype ABR114) blotted onto Hybond‐XL membranes using a 24 × 3 slot apparatus (Schleicher & Schuell, now Cytiva, Germany). A series of dilutions of plasmid 18S rDNA inserts with known DNA concentrations were loaded as the amount standards. The 35S rDNA copy number was determined by quantifying the radioactivity of the bands using a phosphorimager.

### FISH on squashed mitotic preparations, image acquisition and analysis

The chromosomes were prepared as described in Jenkins and Hasterok (2007) from meristematic root‐tip cells of 14 *B. hybridum* genotypes. For the preparation of the slides with nuclei isolated from leaf tissue of *B. hybridum* ABR113, fresh, young leaves were washed briefly in PBS buffer, then washed in TRIS buffer (10 mm TRIS–HCl, pH 7.5, 10 mm Na_2_‐EDTA, 100 mm NaCl) for 20 min and chopped with a razor blade in LB01 buffer (15 mm TRIS–HCl, pH 7.5, 2 mm Na_2_‐EDTA, 0.5 mm spermine tetrahydrochloride, 80 mm KCl, 20 mm NaCl, 0.1% Triton X‐100) on ice. The suspension of nuclei was filtered through a mesh filter (pore size of 30 μm), centrifuged (700 g, 4°C, 2 × 3 min) in 0.1% Triton X‐100 in 1 × PBS to remove the chloroplasts and dropped onto microscopic slides that had been cooled to about 0°C. The slides were air‐dried and stored at −20°C until use.

Due to the highly conserved nature of the rDNA coding sequences, a 2.3‐kb *Cla*I subclone of the 25S rDNA of *A. thaliana* (Unfried and Gruendler, 1990) and the clone pTa794, which contained the 5S rRNA gene from wheat (Gerlach and Dyer, 1980), were used as the probes to detect the 35S and 5S rRNA gene clusters. The clones were labelled by nick translation with either tetramethylrhodamine‐5‐dUTP (Roche, https://www.roche.com) or digoxigenin‐11‐dUTP (Roche). Fluorescence *in situ* hybridization (FISH) was performed according to the protocol described by Idziak *et al*. (2011). The precipitated probes were dissolved directly in a hybridization mixture containing 50% deionized formamide and 20% dextran sulphate in 2 × SSC, denatured at 75°C for 10 min and applied to the slides. The preparations were allowed to hybridize at 37°C for 16–20 h. The hybridization signals were detected using green fluorescein isothiocyanate (FITC)‐conjugated anti‐digoxigenin antibodies (Roche) or, in the case of the probes that had been labelled with tetramethylrhodamine‐5‐dUTP, directly visualized. The chromosomes were counterstained with 2.5 μg ml^−1^ 4′,6‐diamidino‐2‐phenylindole (DAPI) in Vectashield (Vector Laboratories, https://vectorlabs.com/).

Images from slides with root‐tip cells were acquired using a Zeiss Axio Imager.Z.2 wide‐field fluorescence microscope (Zeiss, https://www.zeiss.com) equipped with an AxioCam HRm monochromatic camera, while images of the interphase nuclei isolated from *B. hybridum* leaves were acquired using an Olympus FV1000 confocal system (Olympus, https://www.olympus‐global.com). All images were processed using imagej (https://imagej.nih.gov/ij/).

### 35S rDNA expression analysis

The expression levels were analysed using the RT‐CAPS method according to the protocol of Sochorova *et al*. (2017). In brief, total RNA was isolated from fresh leaves that had been collected from 1‐month‐old plants using an RNeasy Plant Mini Kit (Qiagen). Contaminating DNA was removed from the samples using TURBO^™^ DNase (Ambion, now ThermoFisher Scientific). The reverse transcription reactions (20 μl) typically contained 1 μg of total RNA and 200 units of PrimeScript reverse transcriptase (Takara, https://www.takarabio.com), and were performed using the conditions recommended by the supplier. For ITS1 region amplification using PCR, 1 µl of cDNA and primers 18Sfor and 5.8Srev (Kovarik *et al*., 2005) were used. The ITS1 PCR products were digested with a *Mlu*I restriction enzyme in an amplification mixture. For each, a control PCR, with gDNA as the template, followed by the *Mlu*I restriction of the ITS1 PCR product was performed.

## AUTHOR CONTRIBUTIONS

All of the authors contributed their intellectual input and assistance to this study. NB‐Z, AK and RH conceived of and designed the study. NB‐Z, AK and ER performed the experiments. SG and JV provided the NGS data. NB‐Z, AK, ER and RH analysed the data. MT, GST and RH collected the plant material from Turkey. NB‐Z, RH, AK and JV acquired the financial support. NB‐Z, ER and RH wrote the article, which was edited and approved by all of the authors.

## CONFLICT OF INTEREST

The authors declare no conflicts of interest.

## Supporting information


**Figure S1.** The quantity of 35S rDNA in the genomes of *Brachypodium distachyon* and *Brachypodium stacei* that were analysed by slot‐blot hybridization.
**Figure S2.** Alignment of 222 50‐bp‐long ITS1 variants from *Brachypodium hybridum* ABR113, based upon which the neighbour‐joining phylogenetic tree in Figure 3(b) was constructed.
**Figure S3.** The distribution of the 35S and 5S rDNA loci in the mitotic metaphase chromosomes and interphase nuclei of the *Brachypodium hybridum* ABR101 genotype.
**Figure S4.** The distribution of the 35S and 5S rDNA loci in the mitotic metaphase chromosomes and interphase nuclei of the *Brachypodium hybridum* ABR100 genotype.
**Figure S5.** The distribution of the 35S and 5S rDNA loci in the mitotic metaphase chromosomes and interphase nuclei of the *Brachypodium hybridum* ABR107 genotype.
**Figure S6.** The distribution of the 35S and 5S rDNA loci in the mitotic metaphase chromosomes and interphase nuclei of the *Brachypodium hybridum* ABR115 genotype.
**Figure S7.** The distribution of the 35S and 5S rDNA loci in the mitotic metaphase chromosomes and interphase nuclei of the *Brachypodium hybridum* ABR137 genotype.
**Figure S8.** The distribution of the 35S and 5S rDNA loci in the mitotic metaphase chromosomes and interphase nuclei of the *Brachypodium hybridum* 20‐15 genotype.
**Figure S9.** The distribution of the 35S and 5S rDNA loci in the mitotic metaphase chromosomes and interphase nuclei of the *Brachypodium hybridum* 19‐13‐2 genotype.
**Figure S10.** The distribution of the 35S and 5S rDNA loci in the mitotic metaphase chromosomes and interphase nuclei of the *Brachypodium hybridum* 19‐6‐2 genotype.
**Figure S11.** The distribution of the 35S and 5S rDNA loci in the mitotic metaphase chromosomes and interphase nuclei of the *Brachypodium hybridum* 18‐15 genotype.
**Figure S12.** The distribution of the 35S and 5S rDNA loci in the mitotic metaphase chromosomes and interphase nuclei of the *Brachypodium hybridum* 18‐19 genotype.
**Figure S13.** The distribution of the 35S and 5S rDNA loci in the mitotic metaphase chromosomes and interphase nuclei of the *Brachypodium hybridum* 3‐4‐2 genotype.
**Figure S14.** The distribution of the 35S and 5S rDNA loci in the mitotic metaphase chromosomes and interphase nuclei of the *Brachypodium hybridum* 11‐8 genotype.
**Figure S15.** The distribution of the 35S and 5S rDNA loci in the mitotic metaphase chromosomes and interphase nuclei of the *Brachypodium hybridum* 18‐8‐1 genotype.
**Figure S16.** The distribution of the 35S and 5S rDNA loci in the mitotic metaphase chromosomes and interphase nuclei of the *Brachypodium hybridum* 11‐1‐1 genotype.
**Figure S17.** The distribution of the 35S rDNA loci in the nuclei isolated from leaves of the *Brachypodium hybridum* ABR113 genotype.

**Table S1.** The distribution of high‐frequency SNPs (≥10%) in the 35S rDNA sequences of the *Brachypodium distachyon* genotype ABR5.

**Table S2.** The distribution of low‐frequency SNPs (≥1%) in the 35S rDNA sequences of the *Brachypodium distachyon* genotype ABR5.

**Table S3.** The distribution of high‐frequency SNPs (≥10%) in the 35S rDNA sequences of the *Brachypodium distachyon* genotype Bd21.

**Table S4.** The distribution of low‐frequency SNPs (≥1%) in the 35S rDNA sequences of the *Brachypodium distachyon* genotype Bd21.

**Table S5.** The distribution of high‐frequency SNPs (≥10%) in the 35S rDNA sequences of the *Brachypodium stacei* genotype ABR114.

**Table S6.** The distribution of low‐frequency SNPs (≥1%) in the 35S rDNA sequences of the *Brachypodium stacei* genotype ABR114.

## Data Availability

All relevant data can be found within the article and its supporting materials.
