# Supplementary material for: The fate of 35S rRNA genes in the allotetraploid grass Brachypodium hybridum
Source: Plant J. 2020 Jul 3;103(5):1810–25. doi: 10.1111/tpj.14869 (PMC7497271; doi:10.1111/tpj.14869)
Supplement: Supplementary file 1 — Figure S1. The quantity of 35S rDNA in the genomes of Brachypodium distachyon and Brachypodium stacei that were analysed by slot‐blot hybridization. Figure S2. Alignment of 222 50‐bp‐long ITS1 variants from Brachypodium hybridum ABR113, based upon which the neighbour‐joining phylogenetic tree in Figure 3(b) was constructed. Figure S3. The distribution of the 35S and 5S rDNA loci in the mitotic metaphase chromosomes and interphase nuclei of the Brachypodium hybridum ABR101 genotype. Figure S4. The distribution of the 35S and 5S rDNA loci in the mitotic metaphase chromosomes and interphase nuclei of the Brachypodium hybridum ABR100 genotype. Figure S5. The distribution of the 35S and 5S rDNA loci in the mitotic metaphase chromosomes and interphase nuclei of the Brachypodium hybridum ABR107 genotype. Figure S6. The distribution of the 35S and 5S rDNA loci in the mitotic metaphase chromosomes and interphase nuclei of the Brachypodium hybridum ABR115 genotype. Figure S7. The distribution of the 35S and 5S rDNA loci in the mitotic metaphase chromosomes and interphase nuclei of the Brachypodium hybridum ABR137 genotype. Figure S8. The distribution of the 35S and 5S rDNA loci in the mitotic metaphase chromosomes and interphase nuclei of the Brachypodium hybridum 20‐15 genotype. Figure S9. The distribution of the 35S and 5S rDNA loci in the mitotic metaphase chromosomes and interphase nuclei of the Brachypodium hybridum 19‐13‐2 genotype. Figure S10. The distribution of the 35S and 5S rDNA loci in the mitotic metaphase chromosomes and interphase nuclei of the Brachypodium hybridum 19‐6‐2 genotype. Figure S11. The distribution of the 35S and 5S rDNA loci in the mitotic metaphase chromosomes and interphase nuclei of the Brachypodium hybridum 18‐15 genotype. Figure S12. The distribution of the 35S and 5S rDNA loci in the mitotic metaphase chromosomes and interphase nuclei of the Brachypodium hybridum 18‐19 genotype. Figure S13. The distribution of the 35S and 5S rDNA loci in the [file TPJ-103-1810-s001.docx]

## Supporting Information

Article title: The fate of 35S rRNA genes in the allotetraploid grass *Brachypodium hybridum*

Authors: Natalia Borowska-Zuchowska, Ales Kovarik, Ewa Robaszkiewicz, Metin Tuna, Gülsemin Savaş Tuna, Sean Gordon, John P. Vogel, Robert Hasterok

The following Supporting Information is available for this article:

**Figure S1.** The amount of 35S rDNA in the genomes of *B. distachyon* and *B. stacei* that were analysed by slot-blot hybridisation. The upper panel: Series of dilutions of the genomic DNA that hybridised to 18S rDNA probe. The lower panel: Series of dilutions of the plasmids that contained the 18S rDNA subunit of tobacco that was used as a reference to estimate the rDNA copy number in the *Brachypodium* species.

**
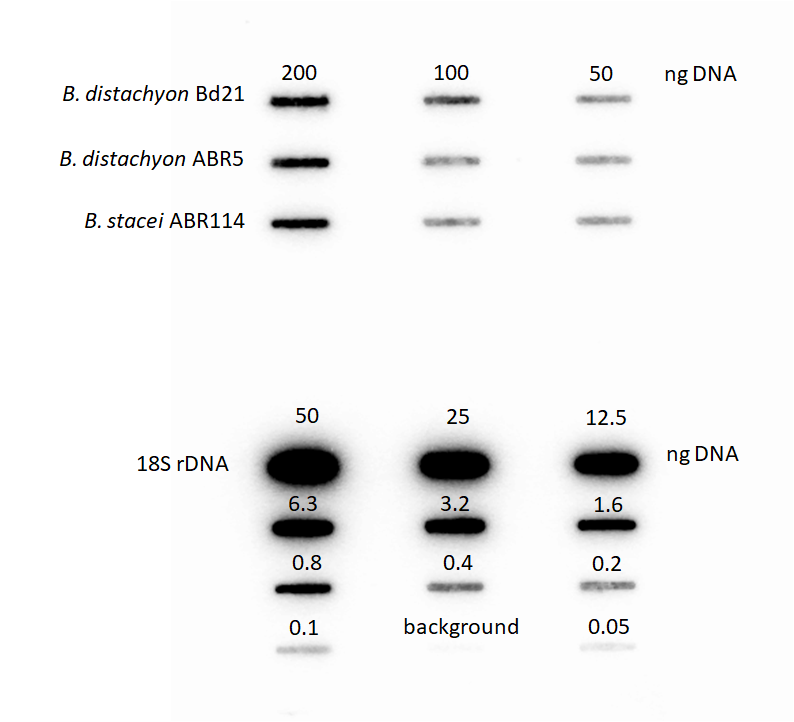
**

**Figure S2.** Alignment of 222 50-bp-long ITS1 variants from *B. hybridum* based on which
a neighbour-joining phylogenetic tree from Fig. 3b was constructed (separate file).

**Figure S3.** The distribution of the 35S and 5S rDNA loci in the mitotic metaphase chromosomes and interphase nuclei of the *B. hybridum* ABR101 genotype. FISH with 25S rDNA (red fluorescence) and 5S rDNA (green fluorescence) as probes on the mitotic metaphase chromosomes (a-d), prometaphase chromosomes with visible secondary constrictions (e-h) and interphase nuclei (i-l). The D-genome and S-genome 35S rDNA loci are indicated as Bd’-Bd” and Bs’-Bs”, respectively. nu – nucleolus. Scale bars: 5 µm.

**
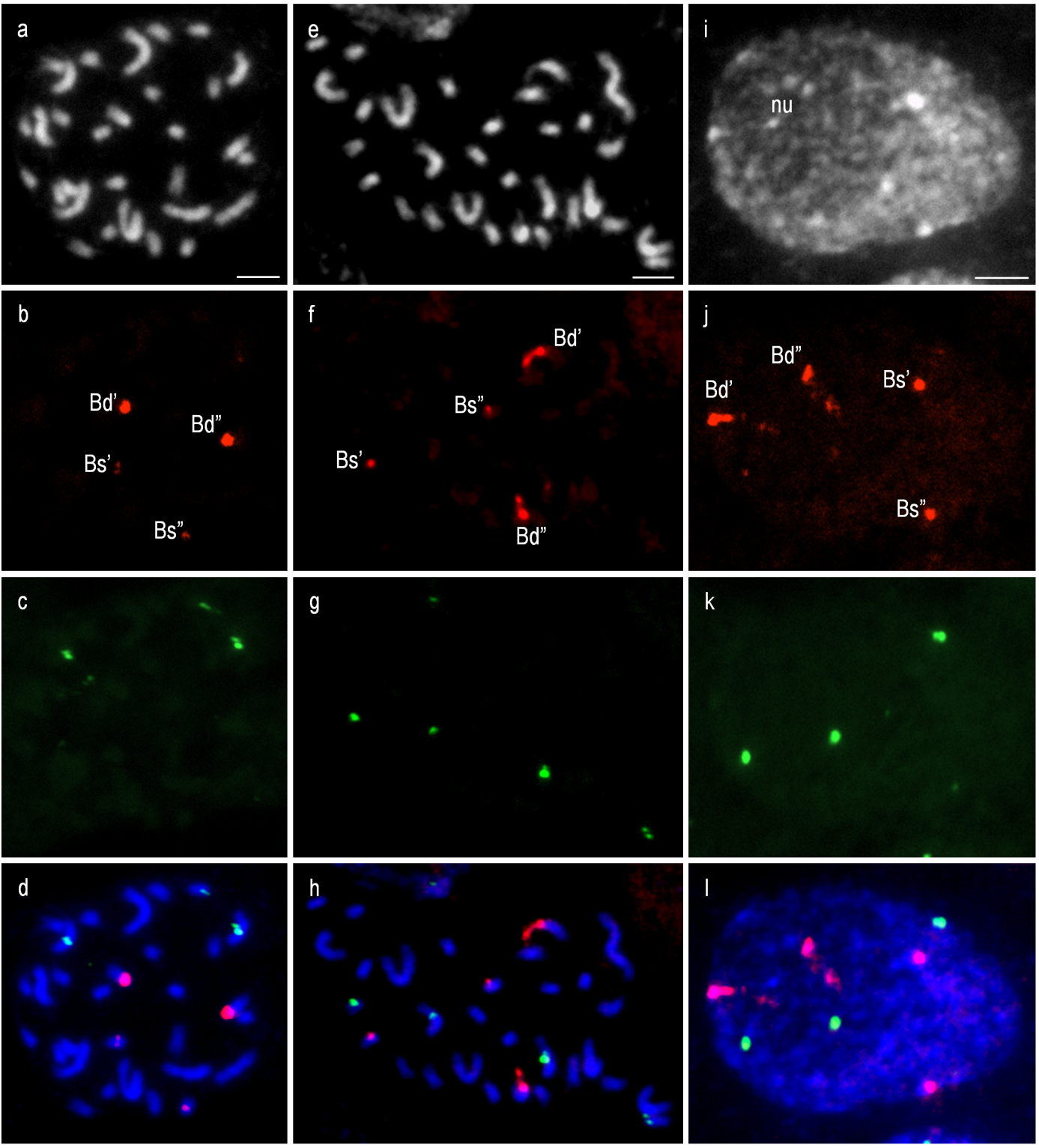
**

**Figure S4.** The distribution of the 35S and 5S rDNA loci in the mitotic metaphase chromosomes and interphase nuclei of the *B. hybridum* ABR100 genotype. FISH with 25S rDNA (red fluorescence) and 5S rDNA (green fluorescence) as probes on the mitotic metaphase chromosomes (a-d), prometaphase chromosomes with visible secondary constrictions (e-h) and interphase nuclei (i-l). The D-genome and S-genome 35S rDNA loci are indicated as Bd’-Bd” and Bs’-Bs”, respectively. nu – nucleolus. dashed lines – decondensed 35S rDNA. Scale bars: 5 µm.

**
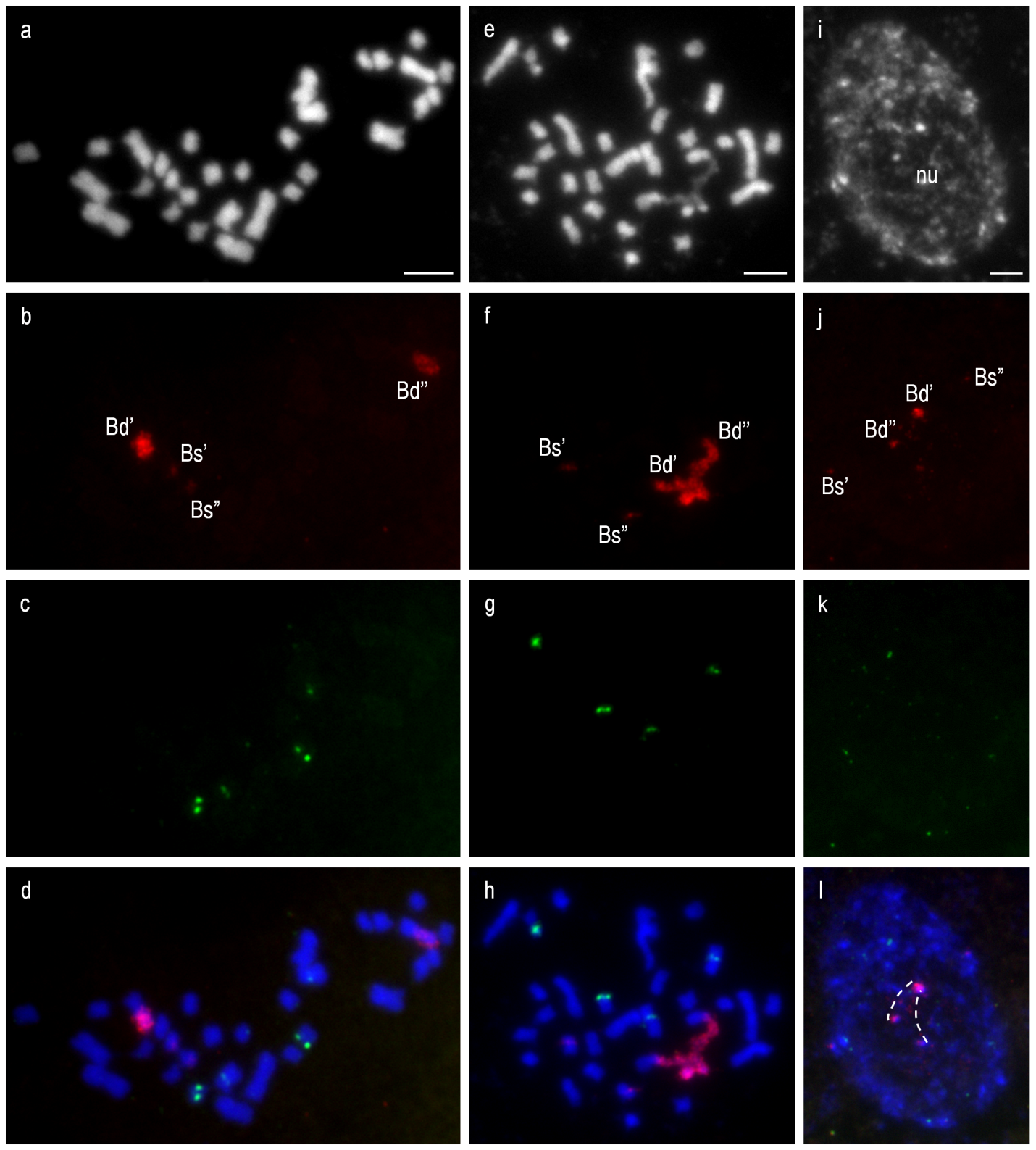
**

**Figure S5.** The distribution of the 35S and 5S rDNA loci in the mitotic metaphase chromosomes and interphase nuclei of the *B. hybridum* ABR107 genotype. FISH with 25S rDNA (red fluorescence) and 5S rDNA (green fluorescence) as probes on the mitotic metaphase chromosomes (a-d), prometaphase chromosomes with visible secondary constrictions (e-h) and interphase nuclei (i-l). The D-genome and S-genome 35S rDNA loci are indicated as Bd’-Bd” and Bs’-Bs”, respectively. nu – nucleolus. Scale bars: 5 µm.

**
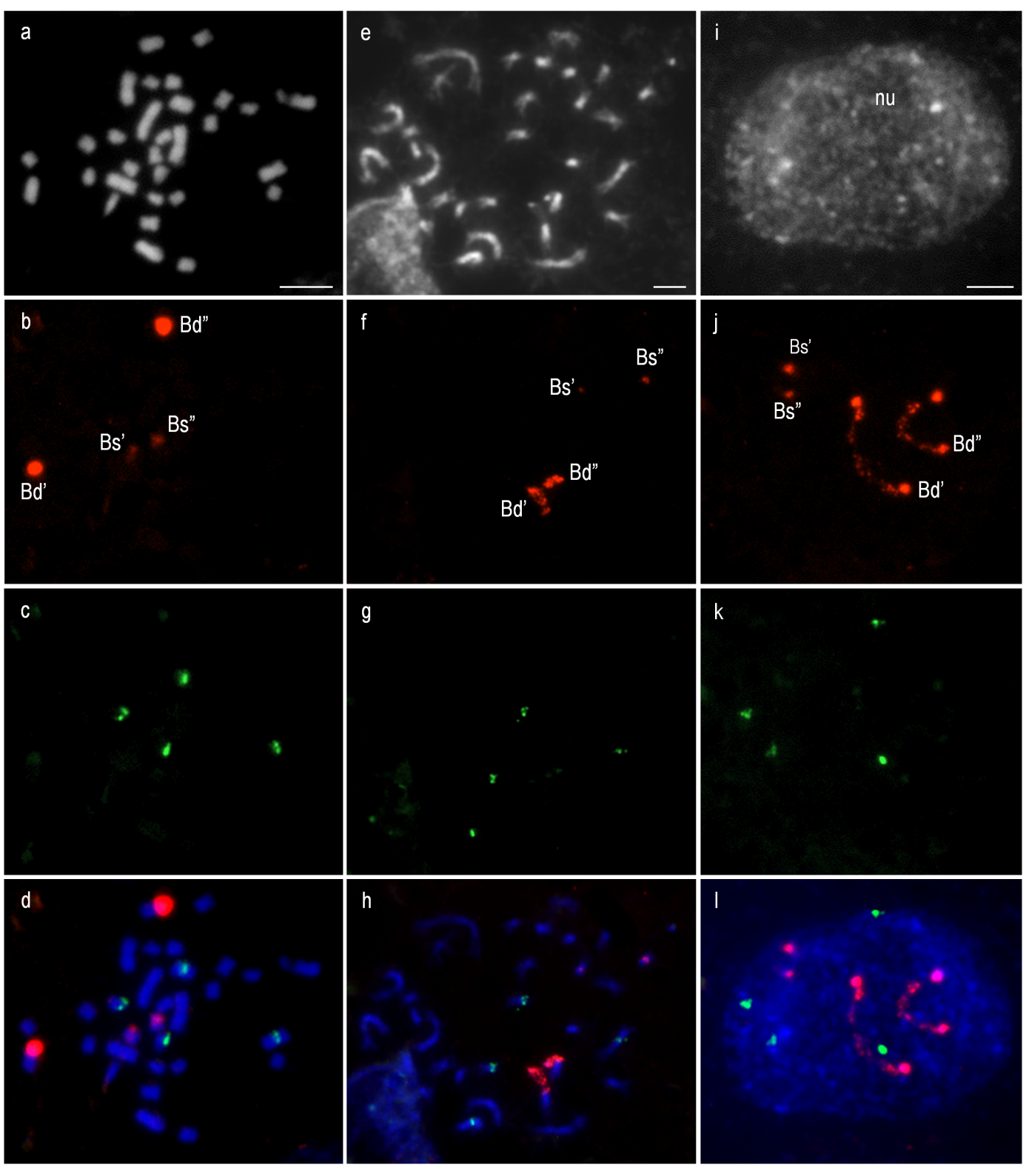
**

**Figure S6.** The distribution of the 35S and 5S rDNA loci in the mitotic metaphase chromosomes and interphase nuclei of the *B. hybridum* ABR115 genotype. FISH with 25S rDNA (red fluorescence) and 5S rDNA (green fluorescence) as probes on the mitotic metaphase chromosomes (a-d), prometaphase chromosomes with visible secondary constrictions (e-h) and interphase nuclei (i-l). The D-genome and S-genome 35S rDNA loci are indicated as Bd’-Bd” and Bs’-Bs”, respectively. nu – nucleolus. dashed lines – decondensed 35S rDNA. Scale bars: 5 µm.

**
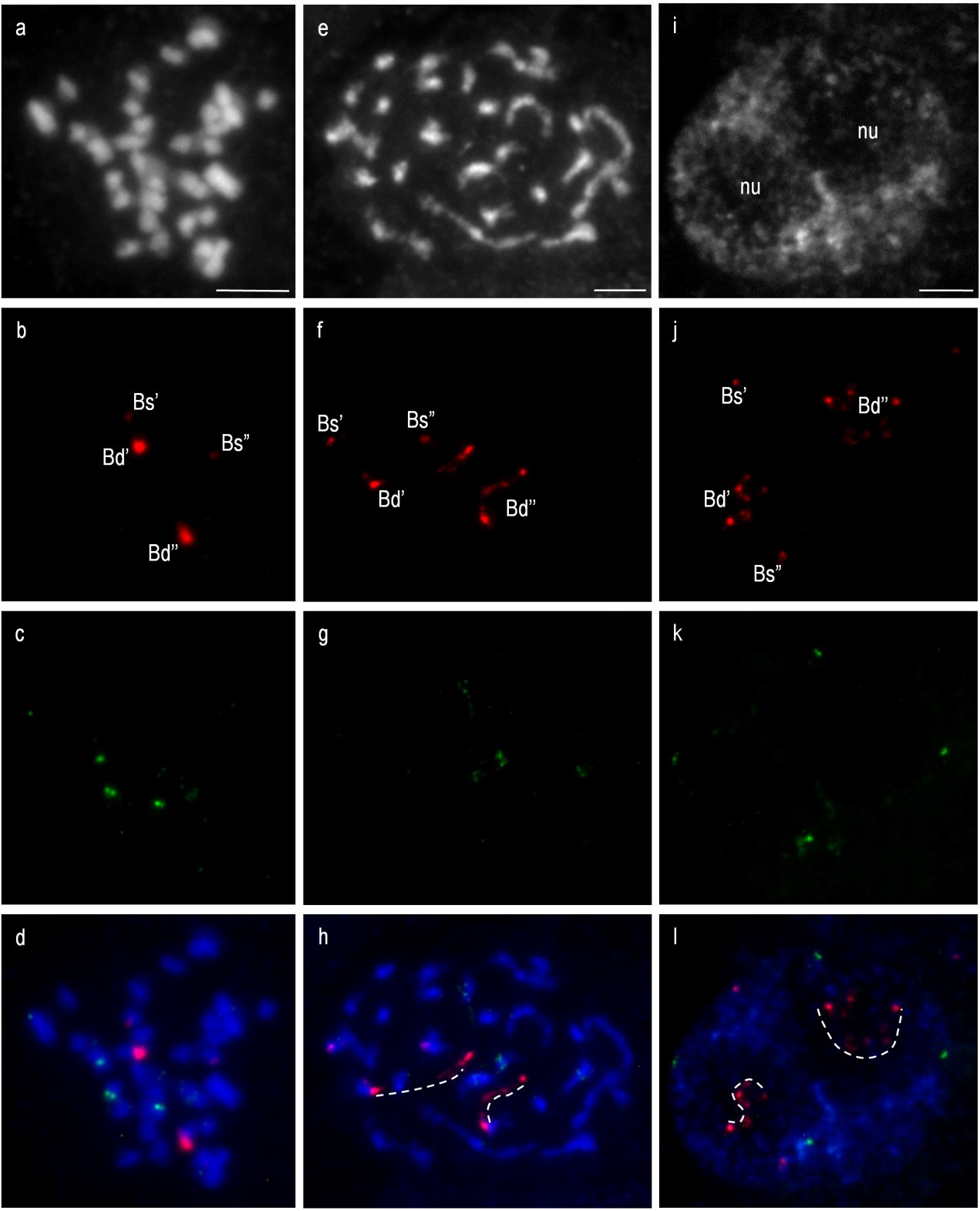
**

**Figure S7.** The distribution of the 35S and 5S rDNA loci in the mitotic metaphase chromosomes and interphase nuclei of the *B. hybridum* ABR137 genotype. FISH with 25S rDNA (red fluorescence) and 5S rDNA (green fluorescence) as probes on the mitotic metaphase chromosomes (a-d), prometaphase chromosomes with visible secondary constrictions (e-h) and interphase nuclei (i-l). The D-genome and S-genome 35S rDNA loci are indicated as Bd’-Bd” and Bs’-Bs”, respectively. nu – nucleolus. Scale bars: 5 µm.

**
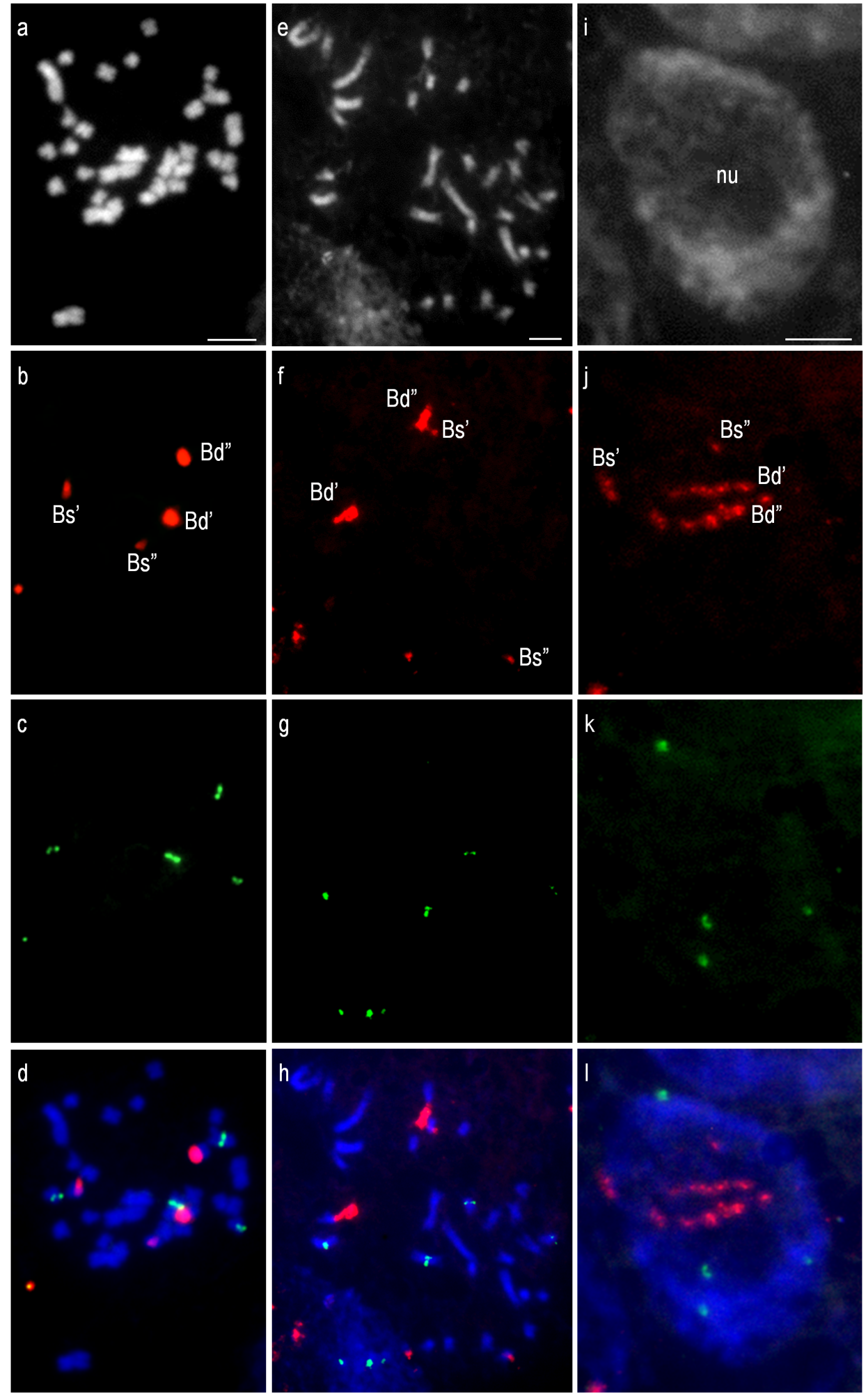
**

**Figure S8.** The distribution of the 35S and 5S rDNA loci in the mitotic metaphase chromosomes and interphase nuclei of the *B. hybridum* 20-15 genotype. FISH with 25S rDNA (red fluorescence) and 5S rDNA (green fluorescence) as probes on the mitotic metaphase chromosomes (a-d), prometaphase chromosomes with visible secondary constrictions (e-h) and interphase nuclei (i-l). The D-genome and S-genome 35S rDNA loci are indicated as Bd’-Bd” and Bs’-Bs”, respectively. nu – nucleolus. dashed lines – decondensed 35S rDNA. Scale bars: 5 µm.

**
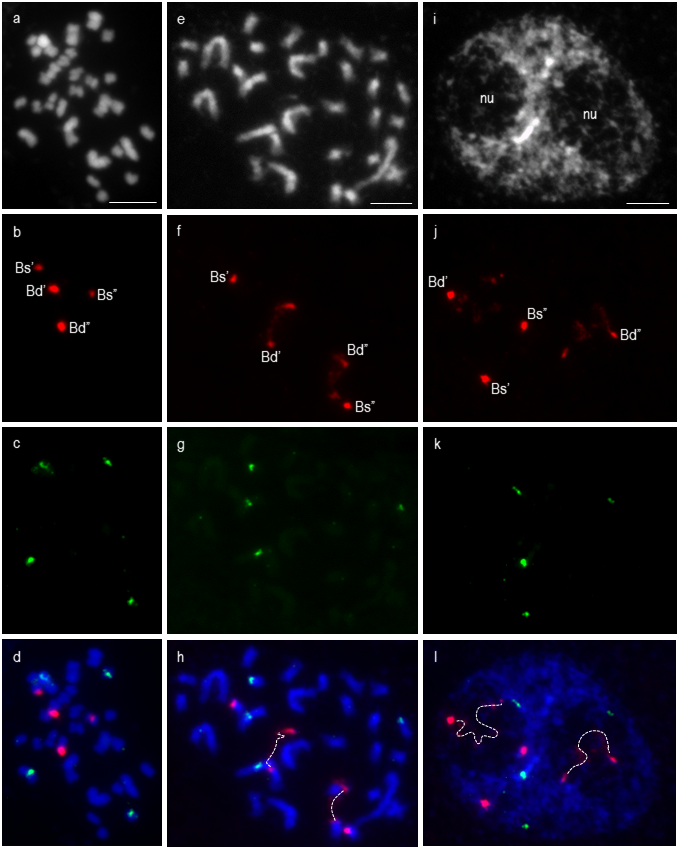
**

**Figure S9.** The distribution of the 35S and 5S rDNA loci in the mitotic metaphase chromosomes and interphase nuclei of the *B. hybridum* 19-13-2 genotype. FISH with 25S rDNA (red fluorescence) and 5S rDNA (green fluorescence) as probes on the mitotic metaphase chromosomes (a-d), prometaphase chromosomes with visible secondary constrictions (e-h) and interphase nuclei (i-l). The D-genome and S-genome 35S rDNA loci are indicated as Bd’-Bd” and Bs’-Bs”, respectively. nu – nucleolus. Scale bars: 5 µm.

**
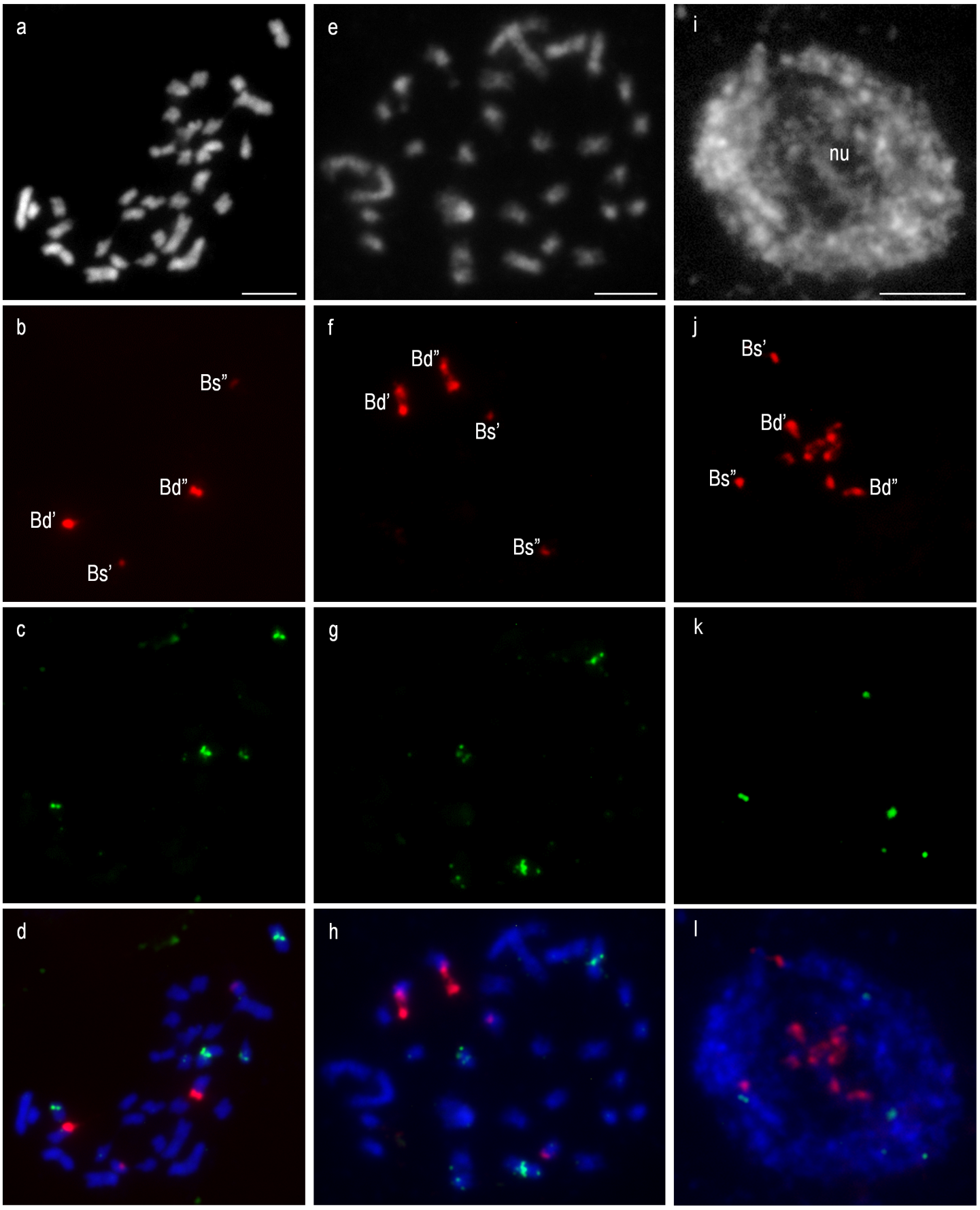
**

**Figure S10.** The distribution of the 35S and 5S rDNA loci in the mitotic metaphase chromosomes and interphase nuclei of the *B. hybridum* 19-6-2 genotype. FISH with 25S rDNA (red fluorescence) and 5S rDNA (green fluorescence) as probes on the mitotic metaphase chromosomes (a-d), prometaphase chromosomes with visible secondary constrictions (e-h) and interphase nuclei (i-l). The D-genome and S-genome 35S rDNA loci are indicated as Bd’-Bd” and Bs’-Bs”, respectively. nu – nucleolus. Scale bars: 5 µm.

**
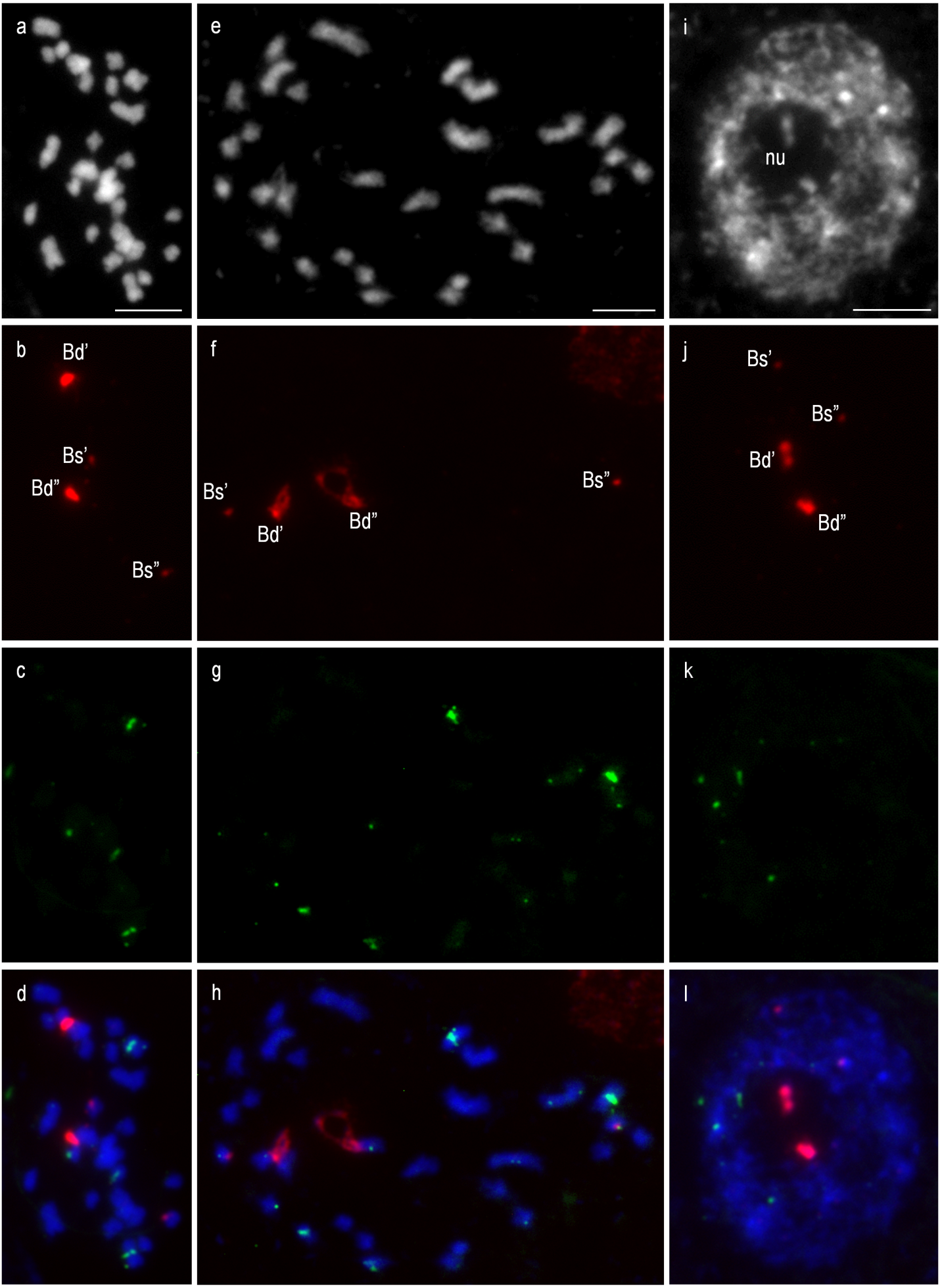
**

**Figure S11.** The distribution of the 35S and 5S rDNA loci in the mitotic metaphase chromosomes and interphase nuclei of the *B. hybridum* 18-15 genotype. FISH with 25S rDNA (red fluorescence) and 5S rDNA (green fluorescence) as probes on the mitotic metaphase chromosomes (a-d), prometaphase chromosomes with visible secondary constrictions (e-h) and interphase nuclei (i-l). The D-genome and S-genome 35S rDNA loci are indicated as Bd’-Bd” and Bs’-Bs”, respectively. nu – nucleolus. Scale bars: 5 µm.


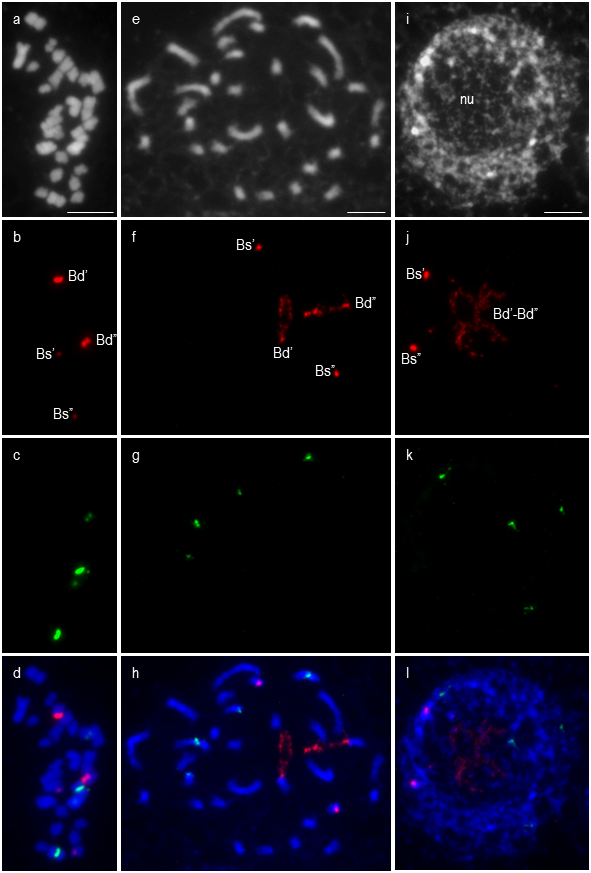


**Figure S12.** The distribution of the 35S and 5S rDNA loci in the mitotic metaphase chromosomes and interphase nuclei of the *B. hybridum* 18-19 genotype. FISH with 25S rDNA (red fluorescence) and 5S rDNA (green fluorescence) as probes on the mitotic metaphase chromosomes (a-d), prometaphase chromosomes with visible secondary constrictions (e-h) and interphase nuclei (i-l). The D-genome and S-genome 35S rDNA loci are indicated as Bd’-Bd” and Bs’-Bs”, respectively. nu – nucleolus. Scale bars: 5 µm.

**
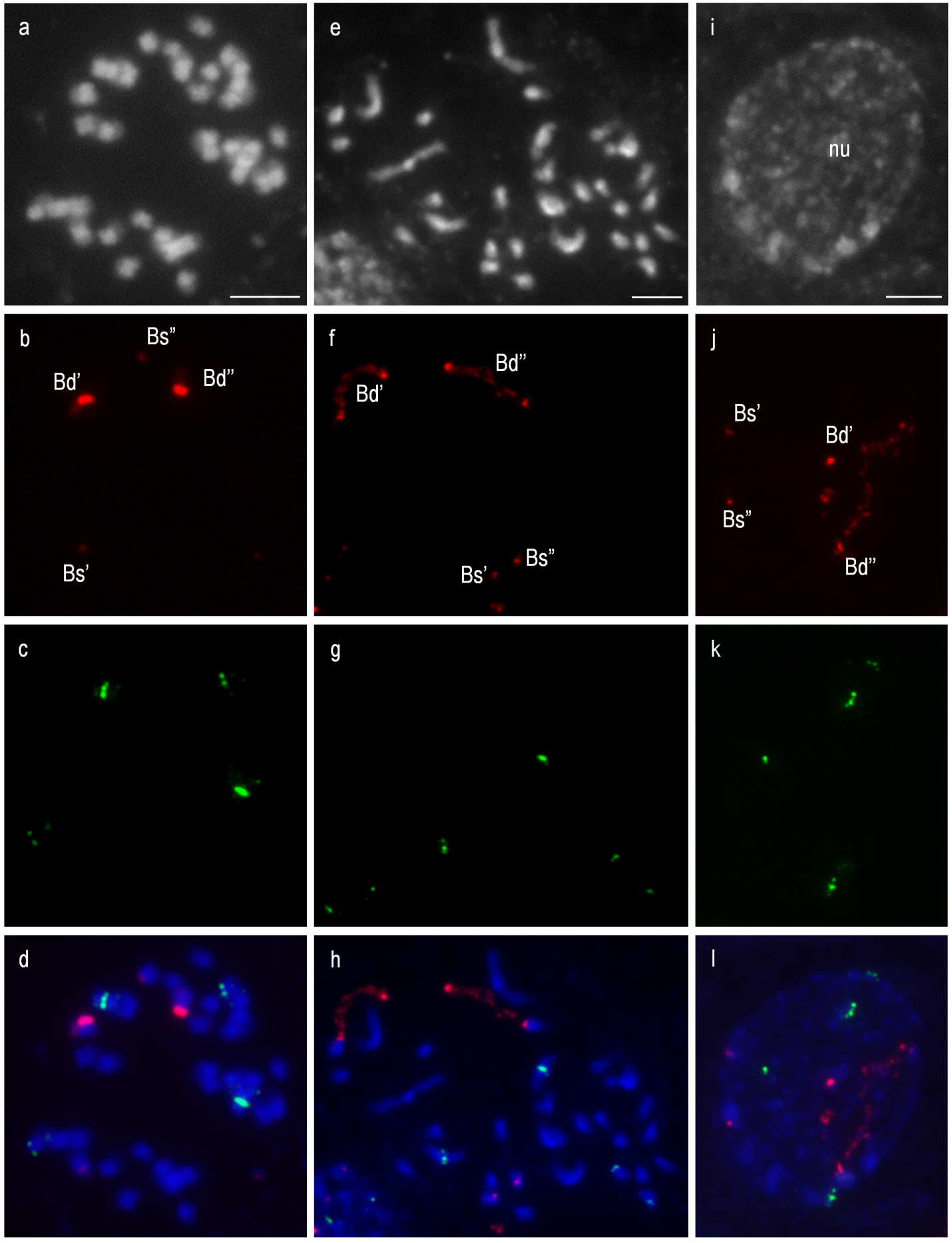
**

**Figure S13.** The distribution of the 35S and 5S rDNA loci in the mitotic metaphase chromosomes and interphase nuclei of the *B. hybridum* 3-4-2 genotype. FISH with 25S rDNA (red fluorescence) and 5S rDNA (green fluorescence) as probes on the mitotic metaphase chromosomes (a-d), prometaphase chromosomes with visible secondary constrictions (e-h) and interphase nuclei (i-l). The D-genome and S-genome 35S rDNA loci are indicated as Bd’-Bd” and Bs’-Bs”, respectively. nu – nucleolus. dashed lines – decondensed 35S rDNA. Scale bars: 5 µm.

**
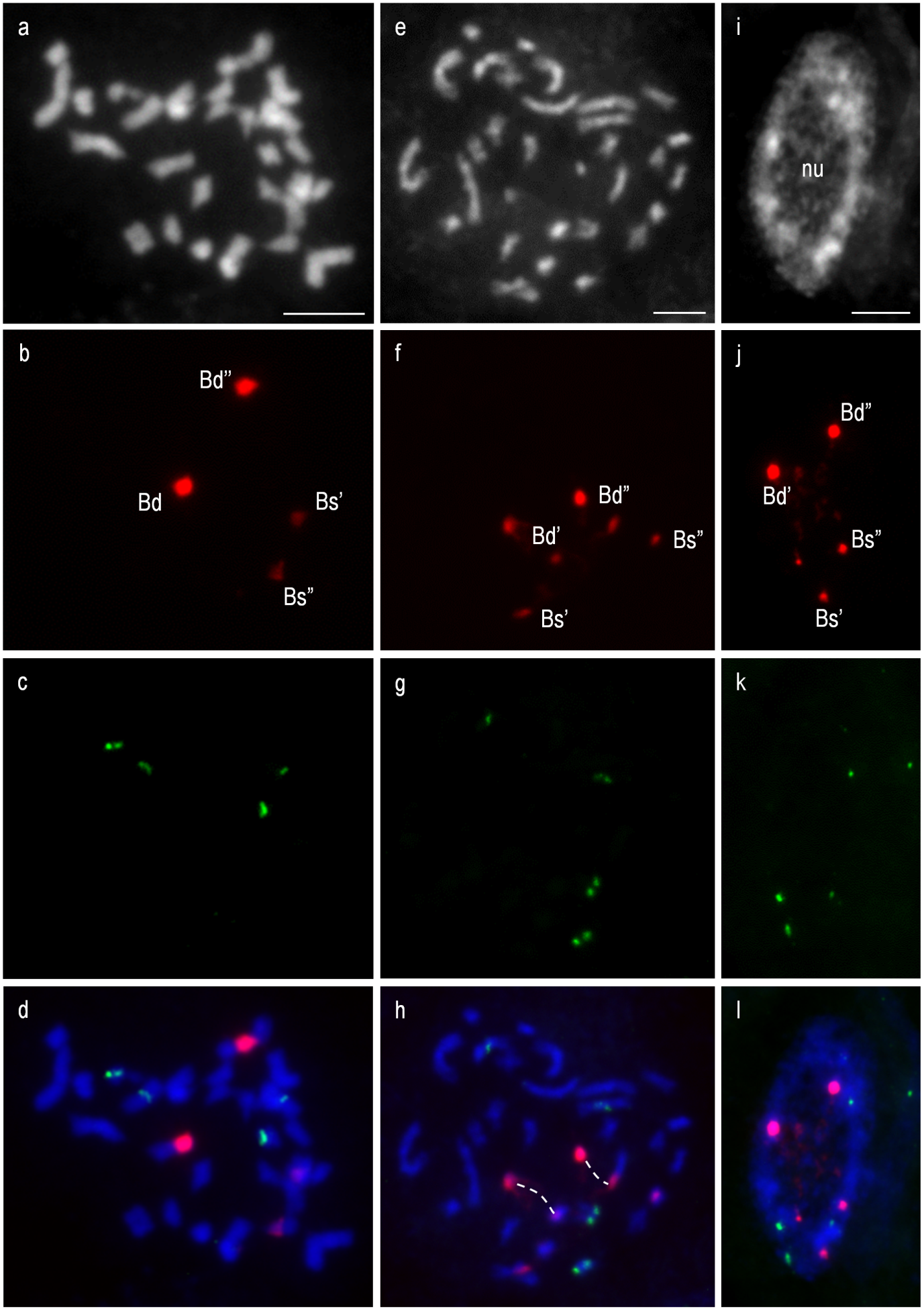
**

**Figure S14.** The distribution of the 35S and 5S rDNA loci in the mitotic metaphase chromosomes and interphase nuclei of the *B. hybridum* 11-8 genotype. FISH with 25S rDNA (red fluorescence) and 5S rDNA (green fluorescence) as probes on the mitotic metaphase chromosomes (a-d), prometaphase chromosomes with visible secondary constrictions (e-h) and interphase nuclei (i-l). The D-genome and S-genome 35S rDNA loci are indicated as Bd’-Bd” and Bs’-Bs”, respectively. nu – nucleolus. Scale bars: 5 µm.

**
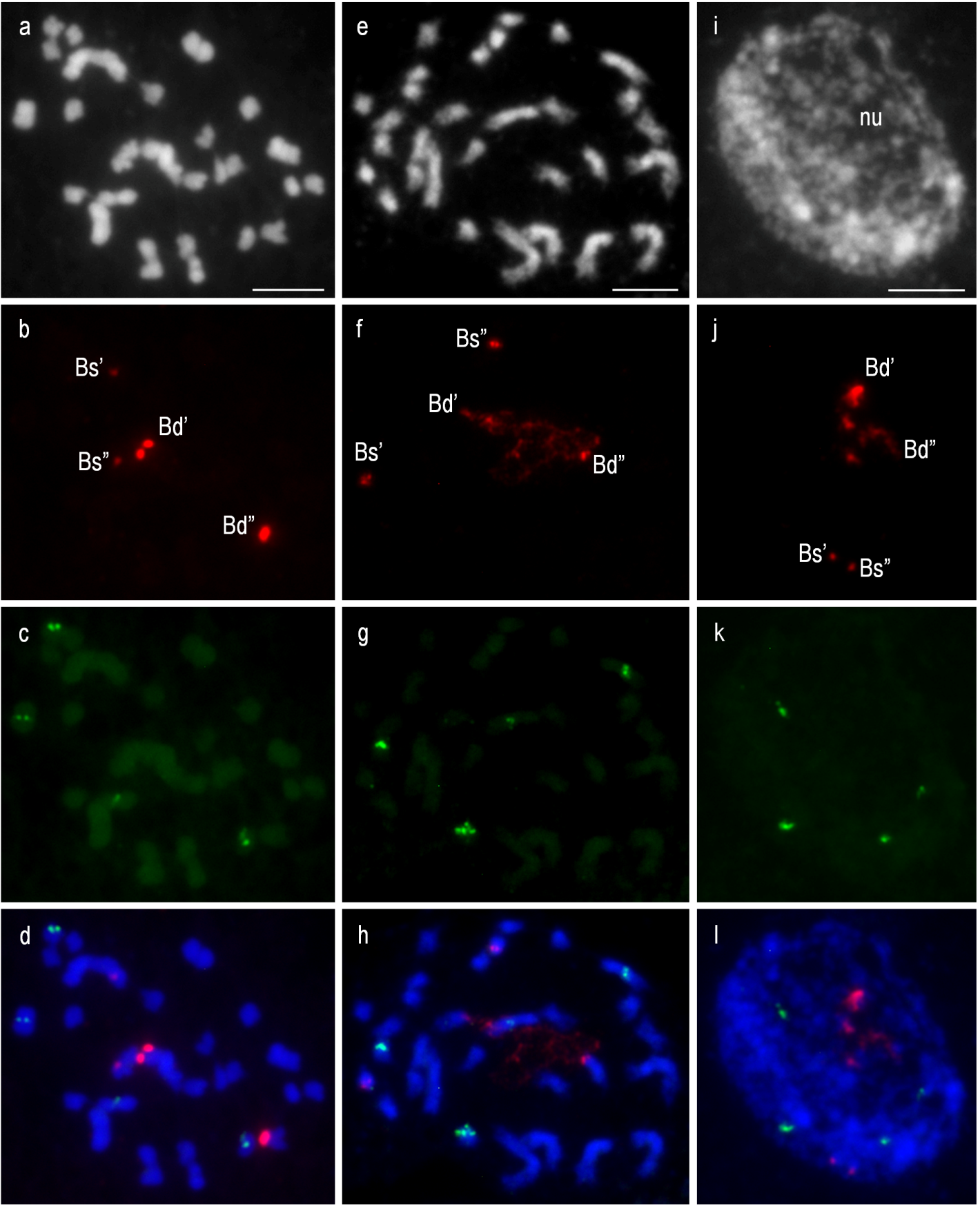
**

**Figure S15.** The distribution of the 35S and 5S rDNA loci in the mitotic metaphase chromosomes and interphase nuclei of the *B. hybridum* 18-8-1 genotype. FISH with 25S rDNA (red fluorescence) and 5S rDNA (green fluorescence) as probes on the mitotic metaphase chromosomes (a-d), prometaphase chromosomes with visible secondary constrictions (e-h) and interphase nuclei (i-l). The D- genome and S-genome 35S rDNA loci are indicated as Bd’-Bd” and Bs’-Bs”, respectively. nu – nucleolus. dashed lines – decondensed 35S rDNA. Scale bars: 5 µm.

**
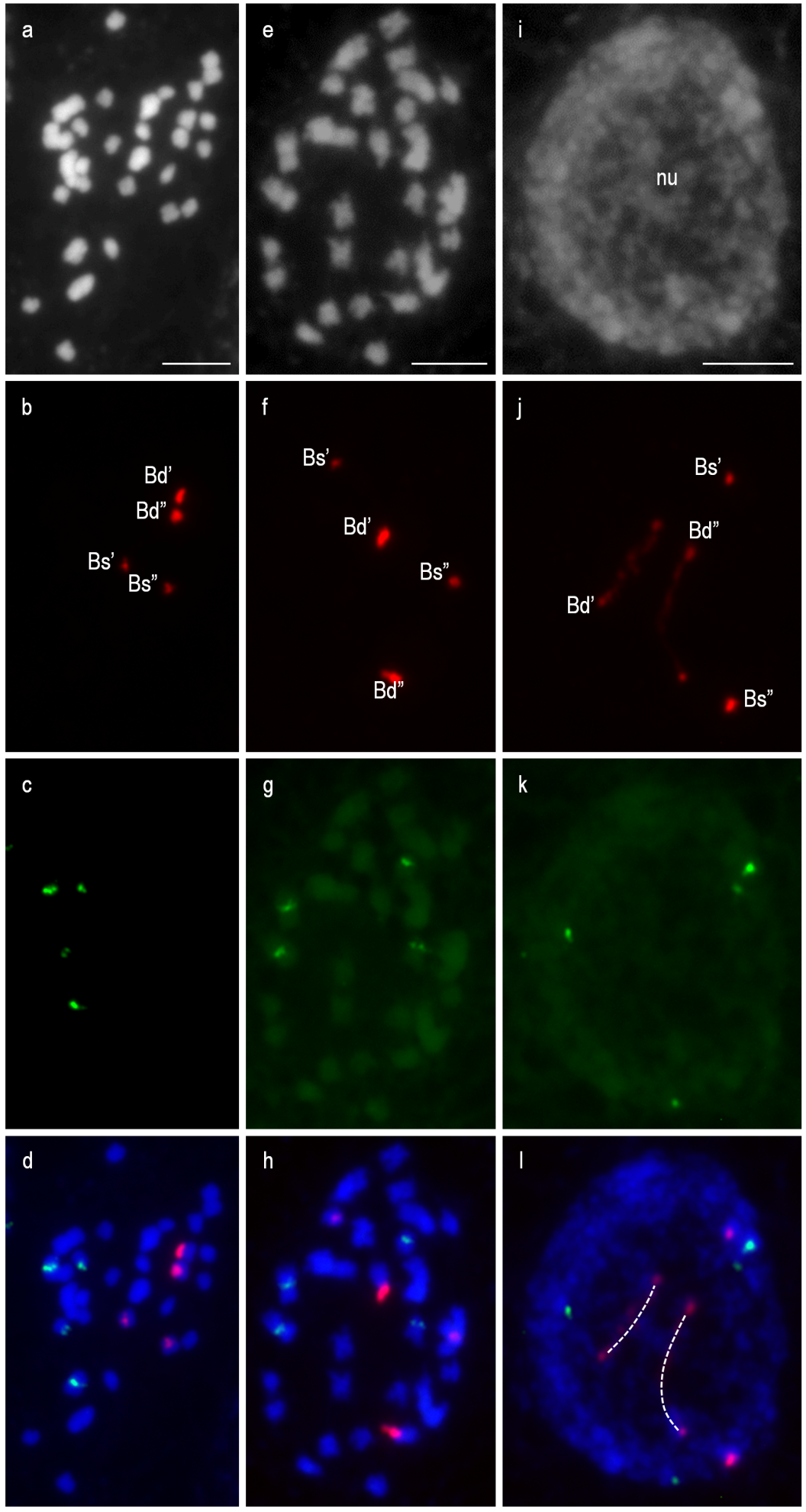
**

**Figure S16.** The distribution of the 35S and 5S rDNA loci in the mitotic metaphase chromosomes and interphase nuclei of the *B. hybridum* 11-1-1 genotype. FISH with 25S rDNA (red fluorescence) and 5S rDNA (green fluorescence) as probes on the mitotic metaphase chromosomes (a-d), prometaphase chromosomes with visible secondary constrictions (e-h) and interphase nuclei (i-l). The D- genome and S-genome 35S rDNA loci are indicated as Bd’-Bd” and Bs’-Bs”, respectively. nu – nucleolus. Scale bars: 5 µm.

**
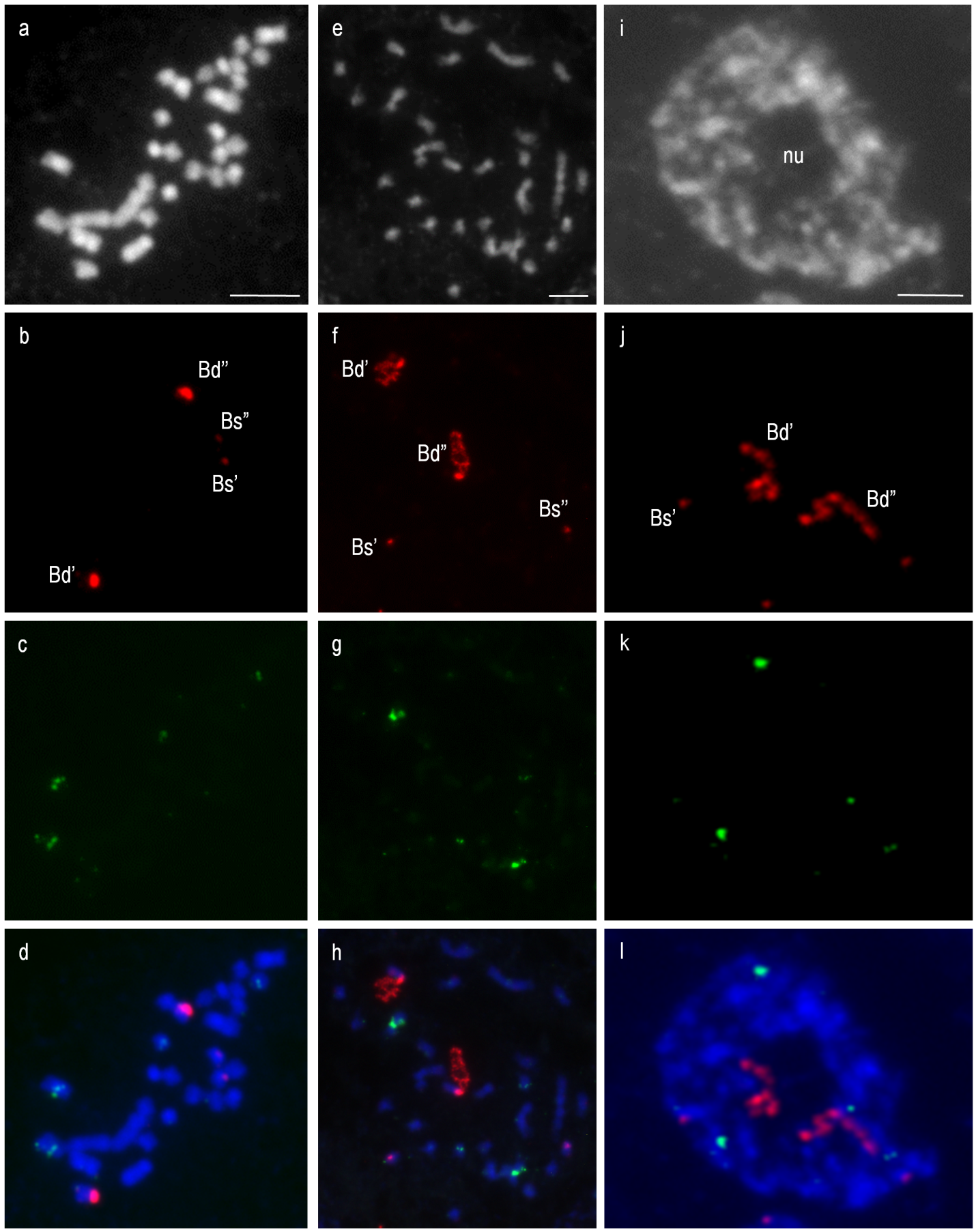
**

**Figure S17.** The distribution of the 35S rDNA loci in the nuclei isolated from leaves of the *B. hybridum* ABR113 genotype. FISH with 25S rDNA (red fluorescence). Six consecutive confocal sections through the representative, DAPI-counterstained nucleus. The D-genome and S-genome 35S rDNA loci are indicated as Bd’-Bd” and Bs’-Bs”, respectively. nu – nucleolus. Scale bar: 5 µm.

**
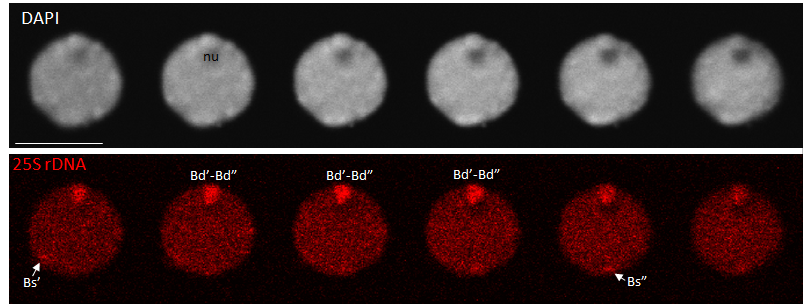
**

**Table S1.** The distribution of high-frequency SNPs (≥10%) in the 35S rDNA sequences of
*B. distachyon* genotype ABR5 (separate Excel file).

**Table S2.** The distribution of low-frequency SNPs (≥1%) in the 35S rDNA sequences of  *B. distachyon* genotype ABR5 (separate Excel file).

**Table S3.** The distribution of high-frequency SNPs (≥10%) in the 35S rDNA sequences of
*B. distachyon* genotype Bd21 (separate Excel file).

**Table S4.** The distribution of low-frequency SNPs (≥1%) in the 35S rDNA sequences of
*B. distachyon* genotype Bd21 (separate Excel file).

**Table S5.** The distribution of high-frequency SNPs (≥10%) in the 35S rDNA sequences of
*B. stacei* genotype ABR114 (separate Excel file).

**Table S6.** The distribution of low-frequency SNPs (≥1%) in the 35S rDNA sequences of
*B. stacei* genotype ABR114 (separate Excel file).
