# Supplementary figures and images for: The fate of 35S rRNA genes in the allotetraploid grass Brachypodium hybridum
Source: Plant J. 2020 Jul 3;103(5):1810–25. doi: 10.1111/tpj.14869 (PMC7497271; doi:10.1111/tpj.14869)

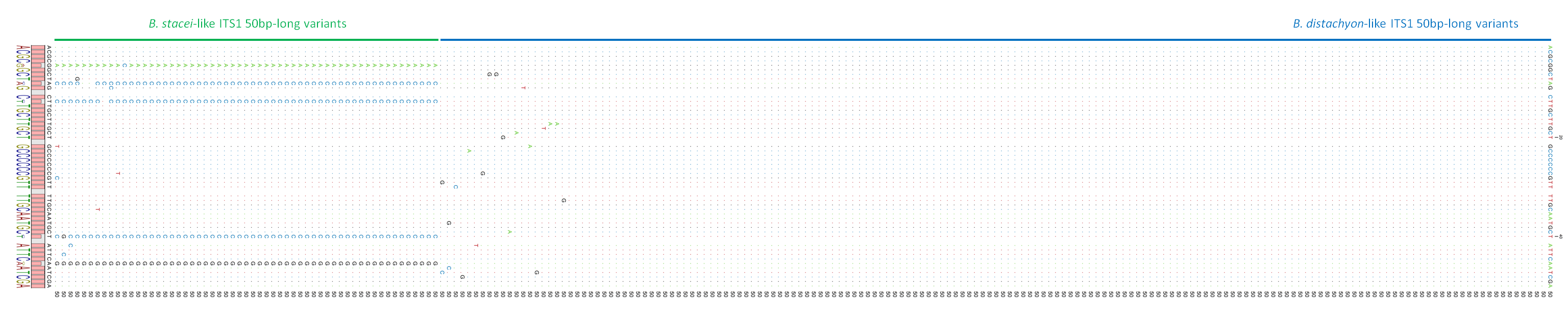

Supplement: Supplementary file 8 [file TPJ-103-1810-s008.tif]
